# Optimization of mTOR Inhibitors Using Property-Based Drug Design and Free–Wilson Analysis for Improved In Vivo Efficacy

**DOI:** 10.1021/acsmedchemlett.3c00351

**Published:** 2023-10-25

**Authors:** Sean T. Murphy, Joy Atienza, Jason W. Brown, Zacharia S. Cheruvallath, Matthew J. Cukierski, Robyn Fabrey, Walter Keung, Lily Kwok, Shawn O’Connell, Mingnam Tang, Darin L. Vanderpool, Patrick W. Vincent, Lilly Zhang, Matthew A. Marx

**Affiliations:** Takeda California, 9625 Towne Centre Drive, San Diego, California 92121, United States

**Keywords:** mTOR, Free−Wilson, property-based drug design, oncology, cancer, kinase inhibitor

## Abstract

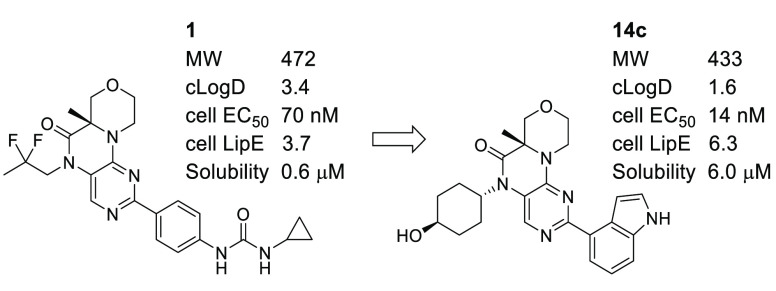

The mTOR kinase regulates a variety of critical cellular processes and has become a target for the treatment of various cancers. Using a combination of property-based drug design and Free–Wilson analysis, we further optimized a series of selective mTOR inhibitors based on the (*S*)-6a-methyl-6a,7,9,10-tetrahydro[1,4]oxazino[3,4-*h*]pteridin-6(5*H*)-one scaffold. Our efforts resulted in **14c**, which showed similar in vivo efficacy compared to previous lead **1** at 1/15 the dose, a result of its improved drug-like properties.

The mammalian target of rapamycin (mTOR)^1^ is a serine/threonine protein kinase and a member of the phosphatidylinositol 3-kinase-related kinase family with sequence similarity to phosphatidylinositol 3-kinases (PI3Ks).^2^ mTOR serves as a core component of two distinct complexes, mTOR complex 1 and mTOR complex 2 (TORC1 and TORC2), each of which regulates different cellular processes.^3^ As an integral part of both complexes, mTOR regulates cell growth, cell survival, cell proliferation, cell motility, protein synthesis, autophagy, and transcription. Due to its involvement in these processes, mTOR inhibitors have been sought for transplant rejection, glycogen storage disease, anticancer, and antiaging.

The extensive mTOR-selective inhibitor literature has been covered by Chen and Zhou up through 2019.^4^ Since then, two reports of CNS-penetrant mTOR inhibitors appeared in 2020 by Bonazzi^5^ and Borsari.^6^ In 2021, a new chemotype discovered through virtual screening was reported by Xu and co-workers.^7^ More recently, De Pascale described novel mTOR inhibitors by replacement of morpholine groups with bioisosteres,^8^ and Burnett described selective bisteric inhibitors of TORC1 leading to RMC-5552, which was designed to limit unwanted effects on glucose metabolism from TORC2 inhibition.^9^

Our previously reported inhibitor **1** (Figure 1) was potent against mTOR and selective against the off-target PI3Kα.^10−12^ The target product profile for **1** and the next-generation analogs described here was for a dual inhibitor against both TORC1 and TORC2. The primary cellular assay measured TORC2 activity by monitoring pAKT-S473^13^ in PC-3 cells (cell IC_50_ 70 nM for **1**). TORC1 activity was measured by monitoring pS6K-T389^14^ in the same cell line (cell IC_50_ 49 nM for **1**). While compound **1** showed significant in vivo efficacy at 10 and 30 milligrams of test compound per kilogram of animal weight (mpk) in mouse xenograft models, its cellular potency of 70 nM and rat oral pharmacokinetics (PK) fraction absorbed^15^ of 22% suggested that further optimization was possible. We undertook a campaign to identify analogs that had improved prospects for a lower efficacious dose while retaining the favorable selectivity and safety profile of compound **1**.

**Figure 1:**
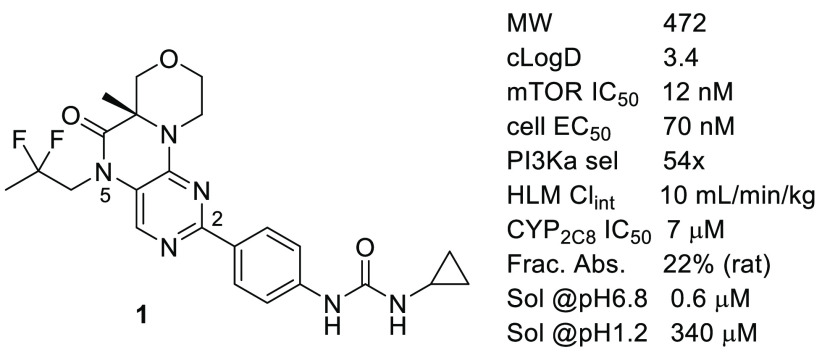
Profile of lead **1**.

We sought to maintain the favorable properties of **1**: selectivity >50× vs PI3Kα, human liver microsome (HLM) intrinsic clearance (Cl_int_) < 10 mL min^–1^ kg^–1^, and cytochrome P450 (CYP) inhibition IC_50_ ≥ 3 μM (for **1**, 2C8 was the most potently inhibited isoform compared to the others tested: 1A2, 2C9, 2C19, 2D6 and 3A4). By modifying the C2 aryl group and the *N5*-alkyl group, we endeavored to improve the in vivo efficacy by improving the cell potency (targeting a 5-fold improvement, i.e., ≤14 nM) and oral absorption (targeting a 3-fold improvement in fraction absorbed, i.e., ≥60%). We also desired to remove the aniline structural alert present in the aryl urea moiety, which has been associated with idiosyncratic adverse drug reactions.^16^ Considering the high calculated log *D* (cLogD)^17^ of 3.4, our primary strategy to maintain low HLM and CYP_2C8_ inhibition while improving oral absorption was to reduce the lipophilicity.

Our modeling of **1** in the mTOR protein^10^ suggested that the two hydrogens in the aniline urea made hydrogen bonds with Asp810, and maintenance of this productive interaction remained a central design theme. A variety of analogs with one hydrogen-bond donor (HBD) and no aniline structural alert were designed. To further lower the lipophilicity, we used the *N5*-cyclopropylmethyl group in **3a** (see Table 1) for our exploration because it had similar cellular potency to **1** and lower cLogD (3.1 vs 3.4). We limited our exploration to analogs with lipophilicity the same or lower than **3a** (i.e., cLogD ≤ 3.1). Although 68 new aryl groups were made and tested in this initial exploration, only two (Table 1, **3b** and **3c**) maintained cell potency comparable to that of the cyclopropylurea aniline **3a**. These new groups were considered promising leads because they eliminated the aniline alert, removed a hydrogen-bond donor, lowered the MW by 58, and reduced the lipophilicity. However, selectivity against PI3Kα was compromised (2- to 4-fold lower selectivity).

**Table 1:**
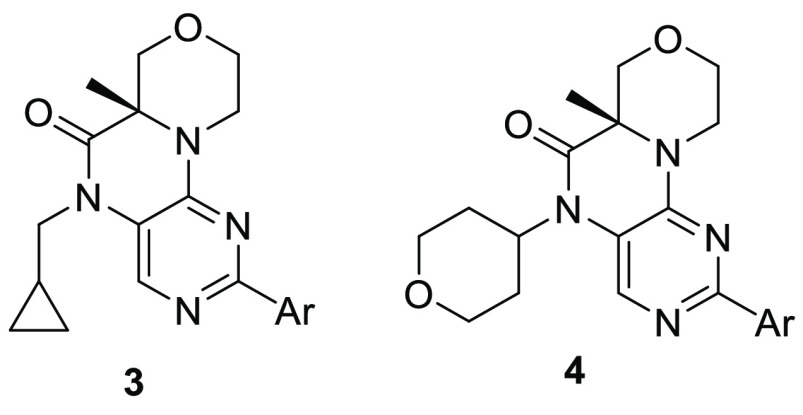

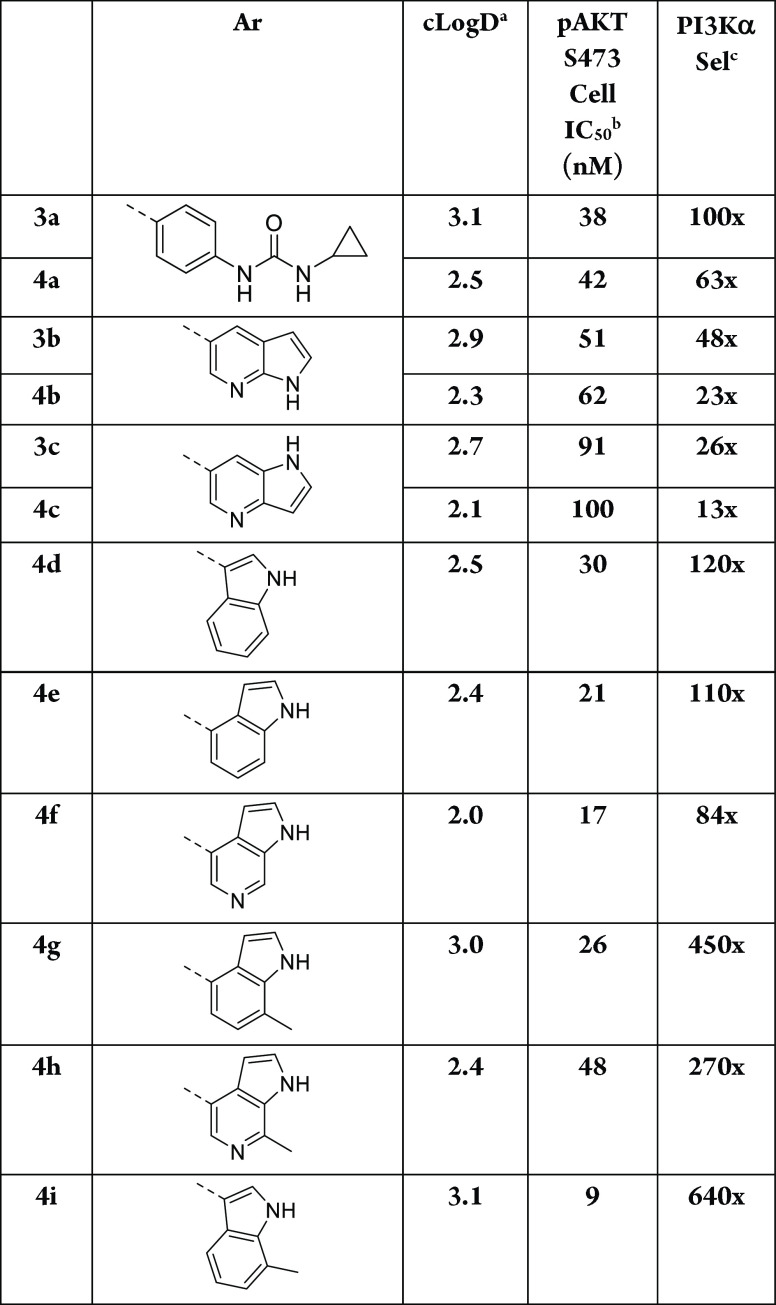
Aniline Urea Replacements

a See ref (17).

b Cellular IC_50_ run in PC-3 cell line measuring p-AKT S473.

c Selectivity is the ratio of the PI3Kα enzyme IC_50_ over the mTOR enzyme IC_50_ as described in ref (10).

To further lower the lipophilicity while maintaining potency, the SAR of the *N5*-alkyl group was studied in the previous aniline urea series. The *N5*-tetrahydropyran (THP) group (structure **4** in Table 1) was found to reduce cLogD by 0.6 units while maintaining potency comparable to the cyclopropylmethyl group (**3a** vs **4a**). Thus, the analogs with the azaindoles were made with the THP group with similar results (**3b** vs **4b** and **3c** vs **4c**). Even though the cell potency was slightly lower in both cases, the gain in lipophilic efficiency (LipE)^18^ was +0.5 for both pairs. The *N5*-THP group thus became the preferred lactam substituent for an expanded exploration of the aryl region.

Because both of the azaindoles connected at the 5- and 6-positions were potent (**4b** and **4c**, respectively), indoles connected at the 3- and 4-positions (Table 1, **4d** and **4e**) were also explored and found to be potent in the cell assay, with IC_50_ ≤ 30 nM. Due to the superior potency and selectivity of the 3- and 4-substituted indoles, many substituted analogs were prepared, and key examples are shown in Table 1. In the 4-indole case, aza substitution in the 6-position was tolerated (**4f**). A methyl group at position 8 maintained potency and gave a dramatic increase in selectivity against PI3Kα (**4g** and **4h**). A similar increase in potency and selectivity resulted from the addition of a methyl group to 3-indole analogue **4d** to give **4i**. While analogue **4i** met our cellular potency criteria, it had a CYP_2C8_ IC_50_ of 1 μM—a result consistent with its higher lipophilicity, which precluded further advancement of this analog.

Compound **4e** was 3-fold more potent than lead compound **1**, and it also met the criteria for selectivity, HLM Cl_int_ (<10 mL min^–1^ kg^–1^), and CYP inhibition (all isoforms >40 μM). We profiled **4e** in rat PK (iv dose of 1 mpk, po dose of 4 mpk in 0.5% methylcellulose) and were pleased to see a %*F* of 91% and a clearance (Cl) of 15 mL min^–1^ kg^–1^, consistent with complete compound absorption.^15^ This was an early indication that the strategy of increasing potency while lowering lipophilicity would allow us to meet our goal of improving prospects for favorable PK and dose.

While **4e** met our HLM and CYP_2C8_ criteria, most of the new analogs did not. We were able to use the information from these early analogs to further refine our desired lipophilicity range to give us the best odds of meeting our goal for a lower efficacious dose. Figure 2 compares the number of compounds that met both the HLM Cl_int_ and CYP_2C8_ criteria (green) to the ones that did not (in red). For the aniline urea analogs made previously, more than half of the analogs passed the criteria only when the cLogD was <2.0. For our initial non-urea analogs, half of the analogs passed the criteria with cLogD < 2.5, which became the design threshold for new analogs.

**Figure 2:**
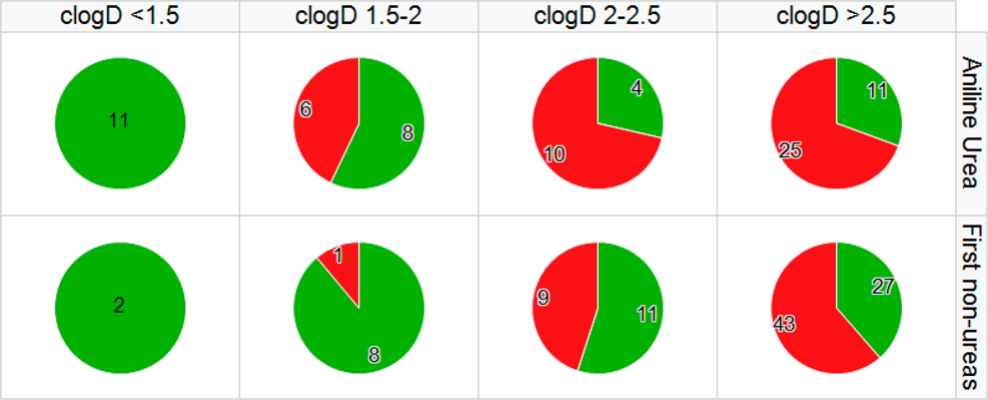
Pie chart analysis of analogs that meet the HLM Cl_int_ and CYP_2C8_ criteria (green) and those that do not (red) considering both cLogD and the *C2*-aryl group type (aniline urea, e.g., **3a**, or non-urea, e.g., **3b**).

The THP group at the 5-position in the aniline urea series was the only branched alkyl group tested previously, and we wanted to further explore these types of branched alkyl groups. However, the existing synthesis (Scheme 1) was very low yielding for most secondary halides and was not feasible for tertiary halides.

**Scheme 1:**
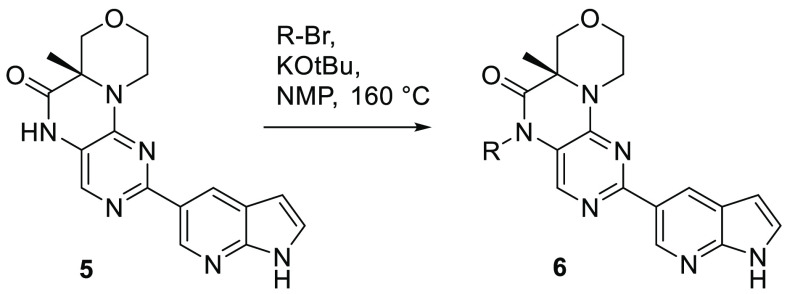
Synthesis of Nonbranched *N5*-Alkyl Groups (R)

To overcome this limitation, we developed the synthesis in Scheme 2, as exemplified for **4e**. The route made use of intermediate **8**, which we had available as the single enantiomer in kilogram quantities from the preclinical development of lead **1**.^19^ After nucleophilic aromatic substitution (S_N_Ar) at the more active chloride of **7** to give pyrimidine chloride **9**, a Suzuki reaction installed the desired aryl group to give **10**. Hydrolysis of the very hindered ester required quite harsh conditions but proceeded to give acid **11** in good yield. A standard amide coupling of the acid to an amine containing the *N5*-alkyl substituent formed intermediate **12**. We were then ready for the key ring closure. Even though the pyrimidine nitrogens are not in a favorable position for S_N_Ar and the electron-donating amino group is deactivating, we were pleased to see that the intramolecular ring closure proceeded in good yield at a modest temperature once the amide was deprotonated with potassium *tert*-butoxide.

**Scheme 2:**
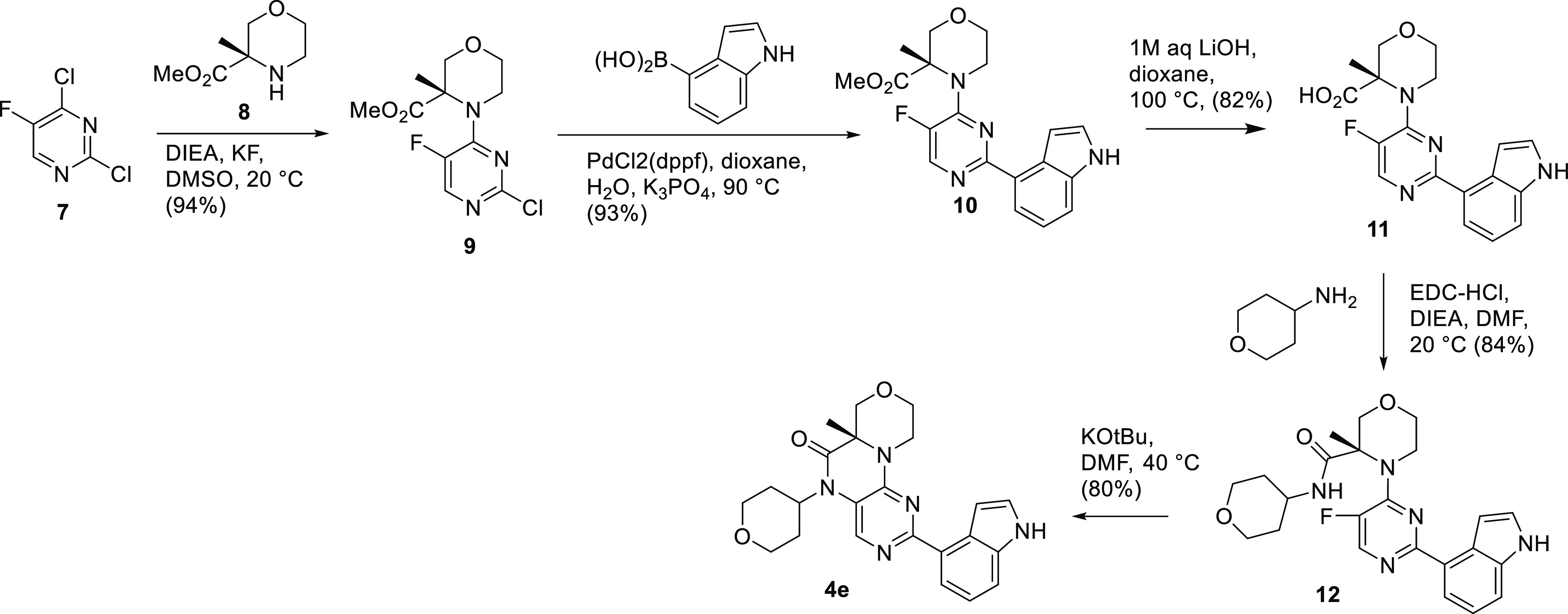
Synthesis of Branched *N5*-Alkyl Groups

With two synthetic routes in hand to explore unbranched and branched substituents, we explored many new *N5*-alkyl groups; the most notable are shown in Table 2. Ring-size reduction of the THP ring in **4b** to the THF ring (**13a**, 2:1 mixture of diastereomers) maintained the cell potency but suffered from reduced selectivity. Opening the THF ring (**13b**) maintained potency and gave a modest increase in selectivity versus PI3Kα but led to increased HLM clearance. Removing the methyl group from **13b** to make **13c** reduced both potency and selectivity but did improve HLM stability, likely from lowering of the lipophilicity, removal of the metabolic liability of O-dealkylation, or both. Because our new synthetic route (Scheme 2) was tailored for branched alkyl groups, we introduced bulky substituents next to N5 in the hopes of improving both potency and selectivity. The most successful was the cyclopropyl analog (**13d**), which gained both potency and selectivity versus **13c**.

**Table 2:**
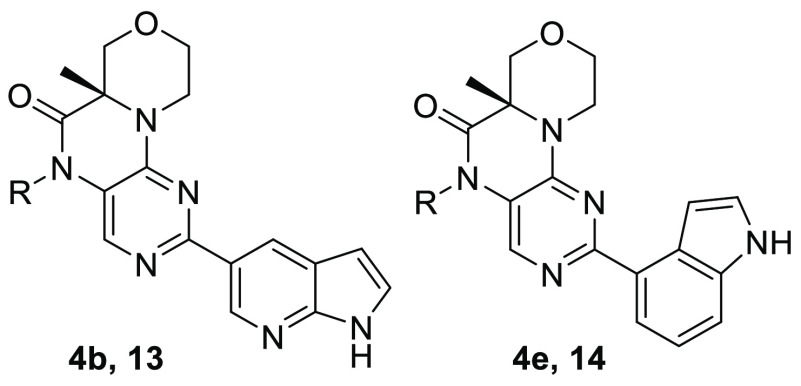

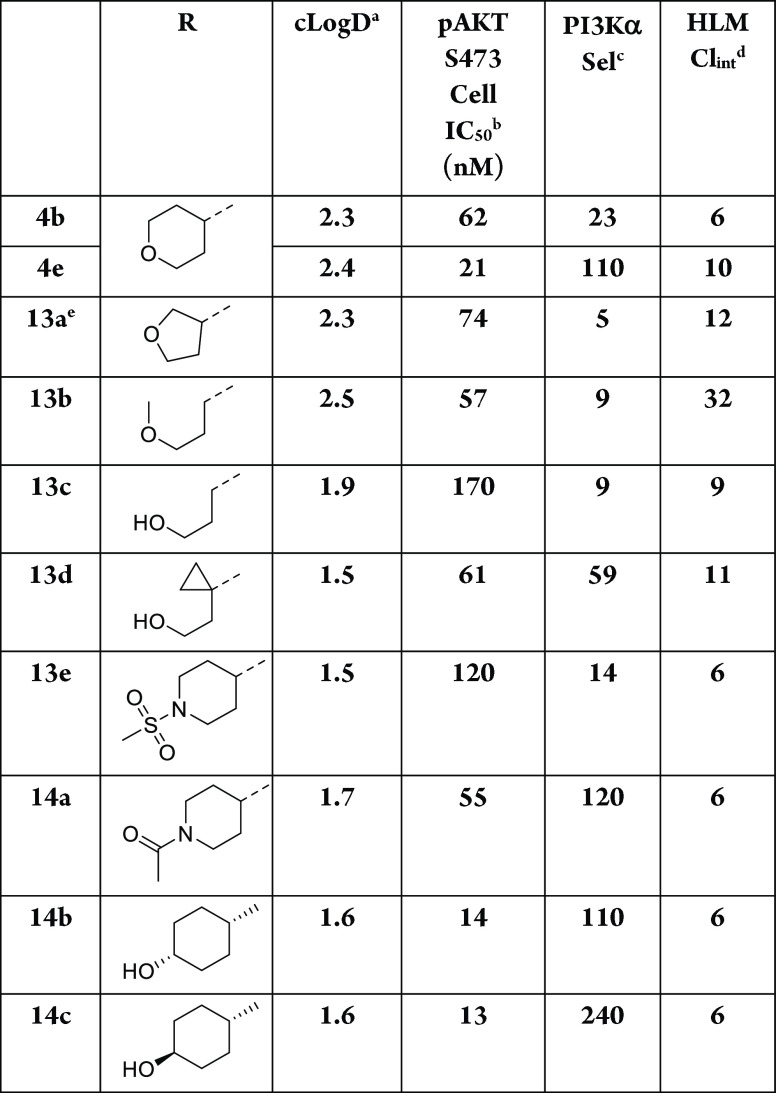
*N5*-Alkyl Group Exploration

a See ref (17).

b Cellular IC_50_ run in PC-3 cell line measuring p-AKT S473.

c Selectivity is the ratio of the PI3Kα enzyme IC_50_ over the mTOR enzyme IC_50_ as described in ref (10).

d HLM apparent intrinsic clearance scaled to units of mL min^–1^ kg^–1^.

e 2:1 mixture of THF diastereomers.

We also further explored six-membered branched alkyl groups (Table 2). Replacing the oxygen in the THP ring with nitrogen and capping with methyl sulfone (**13e**) reduced the potency and selectivity. On the other hand, capping with acetyl (**14a**) maintained potency and improved selectivity. Our first dramatic increase in potency came from the cyclohexanol analogs **14b** and **14c**, which met our cellular potency criterion of ≤14 nM, our selectivity criterion of >50×, and our HLM criterion of <10 mL min^–1^ kg^–1^. Both **14b** and **14c** also showed minimal CYP_2C8_ inhibition (4 and 21 μM, respectively).

For the final round of analog design, we performed a Free–Wilson analysis^20^ on our existing analogs to find new combinations of *C2*-aryl and *N5*-alkyl groups that might also meet our criteria. Free–Wilson analysis assumes additive SAR, and our analysis suggested that the enzyme potencies for both mTOR and PI3Kα were indeed additive. This allowed us to predict not only potency but also selectivity.

The Free–Wilson analysis included the analogs with the substructure shown in Figure 3 and created all possible combinations of R_1_ and R_2_ (>28,000 analogs). In addition to predicting the enzyme potency and selectivity (ratio of predicted PI3Kα potency over predicted mTOR potency), physicochemical properties were also calculated. Analogs were then selected for synthesis according to the criteria shown in Figure 3.

**Figure 3:**
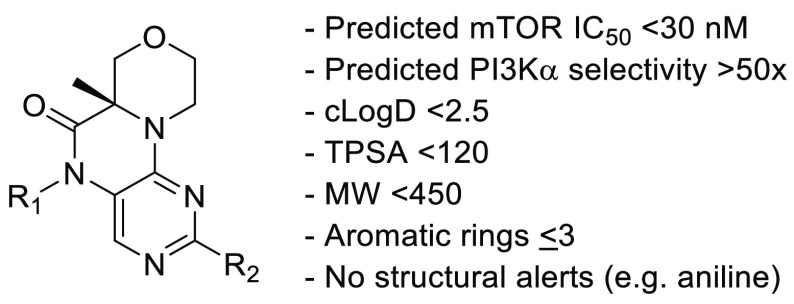
Substructure core used for Free–Wilson analysis and criteria used for selecting analogs for synthesis.

Based on these predictions, a few dozen analogs were synthesized, some of which are shown in Table 3. In general, the actual results were within 2-fold of the prediction or better (i.e., more potent and/or more selective). For the *trans*-cyclohexanol (R_1_ = A), both analogs (**15a** and **15b**) met the potency, selectivity, HLM, and CYP_2C8_ criteria. In the case of the *cis*-cyclohexanol N5 group (R_1_ = B), the analogs met most but not all of the criteria: the 4-indole analog **15c** was not potent enough in the cell assay, and the 3-indole analog **15d** was too potent in the CYP_2C8_ inhibition assay. Finally, the cyclopropyl alcohol group (R_1_ = C) with the 3-indole R_2_ group (**15e**) met all of the criteria except for CYP_2C8_ inhibition.

**Table 3:**
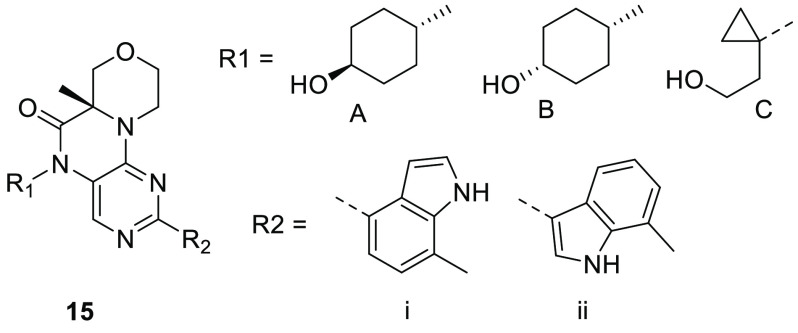
New Combinations from Free–Wilson Analysis

	R_1_	R_2_	mTOR IC_50_/nM (pred)^a^	PI3Ka sel. (pred)^b^	pAKT S473 cell IC_50_/nM	HLM Cl_int_ mL min^–1^ kg^–1^	CYP IC_50_/μM^c^
**15a**	A	i	10 (17)	980× (380×)	12	6	5^d^
**15b**	A	ii	10 (15)	1000× (380×)	7	7	3
**15c**	B	i	12 (25)	800× (220×)	18	8	3
**15d**	B	ii	13 (22)	740× (220×)	9	7	0.8
**15e**	C	ii	7.1 (11)	1400× (1900×)	5	6	1.3

a Predicted mTOR enzyme IC_50_ value from Free–Wilson analysis.

b Predicted selectivity based on the ratio of the predicted PI3Ka enzyme IC_50_ over the predicted mTOR enzyme IC_50_ as described in ref (10).

c The CYP IC_50_ value with the most potent inhibition was 2C8 unless otherwise noted.

d CYP-2C9.

All the analogs that met our in vitro criteria (except for **15b** due to the borderline CYP inhibition) were advanced to rat PK studies to assess the exposure levels and the fraction absorbed (Table 4). Both analogs with the 4-indole and cyclohexanol groups (**14b** and **14c**) had excellent bioavailability and corresponding fraction absorbed as well as low clearance and high exposure. Both analogs met our criteria for the desired improvement over previous lead **1**. The new combination discovered from Free–Wilson analysis (**15a**) showed lower bioavailability and had lower exposure levels. Because the clearance is low for **15a**, the low bioavailability is likely due to poor absorption from low solubility, low permeability, or both.

**Table 4:** Rat PK^a^ on Analogs Meeting In Vitro Criteria

	*C*_max_^b^ (μM)	%*F*	Cl mL min^–1^ kg^–1^	*V*_d_ L/kg	frac. abs.^c^
**1**	0.32	20%	6.9	0.95	22%
**14b**	1.4	100%	11	3.9	100%
**14c**	1.6	94%	7.0	1.3	100%
**15a**	0.57	35%	10	1.6	41%

a iv 1 mpk (*n* = 3), po 4 mpk in 0.5% methylcellulose (*n* = 3).

b po dosing.

c Fraction absorbed estimated from bioavailability (%*F*), Cl, and hepatic blood liver flow (*Q*_h_) = 70 mL min^–1^ kg^–1^.^13^

While **14b** and **14c** had similar cellular potency and rat PK results, analog **14c** was selected for further testing due to its better selectivity versus PI3Kα (240× vs 110×) and lower inhibition of CYP_2C8_ (21 μM vs 4 μM). Analog **14c** showed minimal human ether-a-go-go (hERG) inhibition (patch clamp IC_50_ > 40 μM) and was negative in the Ames test with and without S9 (supernatant fraction obtained from liver homogenate by centrifuging at 9000*g* for 20 min) activation. As specified in the target-product profile, it was also potent against TORC1 (pS6K-T389 cell IC_50_ in PC-3 cells = 6 nM). Selectivity against the other PI3K family members was acceptable: PI3Kβ (180×), PI3Kδ (6×), and PI3Kγ (180×). In a panel of 298 kinases (Invitrogen) tested at 1 μM, only two had inhibition >20%: interleukin-1 receptor-associated kinase 4 (IRAK1) (22%) and cyclin-dependent kinase 9 (CDK9) (34%). While some mTOR active-site inhibitors have shown DNA-PK activity,^21^**14c** showed minimal inhibition (13% at 1 μM, Invitrogen).

Based on the improved rat PK for **14c**, it was dosed at 2 and 6 mpk along with lead **1** at 30 mpk—in an effort to achieve similar in vivo exposure profiles—in a mouse xenograft model with the lung carcinoma cell line A549 (Figure 4). All dosing groups showed less than 20% body weight loss throughout the duration of the study (data in the Supporting Information). Both the 30 mpk dose of **1** and the 2 mpk dose of **14c** fully inhibited tumor growth, with modest tumor burden reduction observed while dosing for 14 days. The 6 mpk dose of **14c** reduced the tumor volume by more than 50%. Regrowth was included to assess a possible growth lag period, but as expected, tumor regrowth resumed following mTOR inhibitor withdrawal.^22^ Analog **14c** showed comparable in vivo efficacy as lead **1** but at 1/15 the dose, which is likely a combination of the higher cellular potency (10 nM vs 70 nM) and improved oral absorption (fraction absorbed 100% vs 20%). The improved absorption is consistent with the 10-fold increase in thermodynamic solubility at pH 6.8 (6.0 μM for **14c** vs 0.6 μM for **1**) and at pH 1.2 (3960 μM for **14c** vs 340 μM for 1).

**Figure 4:**
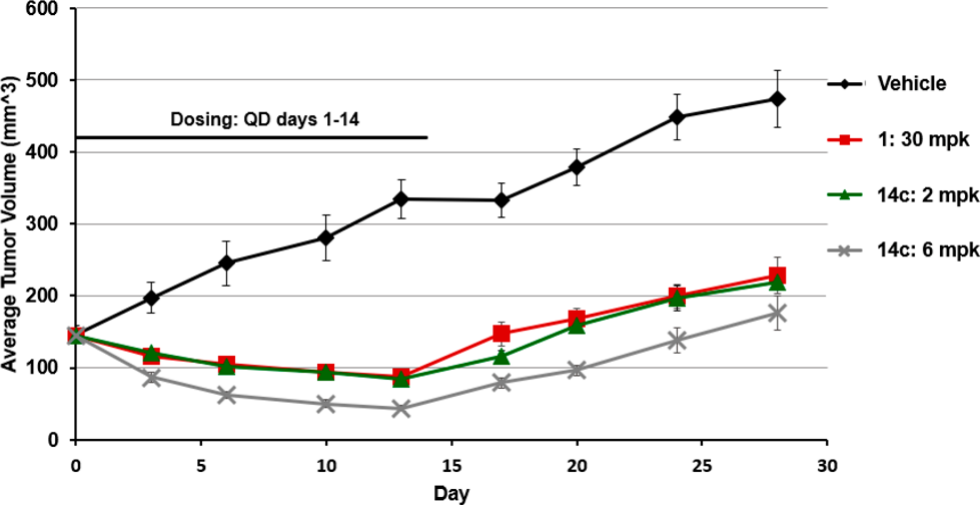
Mouse model with the A549 lung carcinoma cell line.

In summary, we were able to optimize our previous lead **1** to discover **14c** with improved potency, selectivity, and oral absorption. The aniline urea group was replaced with a variety of indoles and azaindoles, which not only removed the structural alert but also lowered the lipophilicity while maintaining or improving potency. A new synthesis was then developed which allowed branched alkyl groups to be explored and led to the discovery of **14c**. Additional analogs were made that met our in vitro criteria by carefully selecting new combinations with the aid of Free–Wilson analysis, which predicted both mTOR potency and selectivity versus PI3Kα. Rat PK results along with in vitro safety considerations led us to test **14c** in our in vivo xenograft model, where it demonstrated equivalent efficacy to our previous lead **1** at 1/15 the dose.

## Supporting Information Available

Synthetic experimental procedures and chemical compound characterization for all previously undisclosed compounds; complete characterization data for key compound **14c**; HPLC purity for in vivo compounds; cellular assay counts and standard deviations; cellular assay protocol; in vivo TGI study design; TGI study body weights (PDF)

## Abbreviations

CDK9: cyclin-dependent kinase 9
Cl: clearance
Cl_int_: intrinsic clearance
cLogD: calculated log *D*
CYP: cytochrome P450
%*F*: bioavailability
HBD: hydrogen-bond donor
hERG: human ether-a-go-go gene
HLM: human liver microsome
IRAK4: interleukin-1 receptor-associated kinase 4
iv: intravenous
LipE: lipophilic efficiency
MM2: molecular mechanics (force field)
mpk: milligrams of test compound per kilogram of animal weight
mTOR: mammalian target of rapamycin
MW: molecular weight
p-AKT: phosphorylated protein kinase B
PI3K: phosphatidylinositol 3-kinase
PK: pharmacokinetics
po: per os (oral)
*Q*_h_: hepatic blood liver flow
S9: supernatant fraction obtained from liver homogenate by centrifuging at 9000*g* for 20 min
SAR: structure–activity relationship
S_N_Ar: nucleophilic aromatic substitution
TGI: tumor growth inhibition
THP: tetrahydropyran
TORC1: mTOR complex 1
TORC2: mTOR complex 2
TPSA: topological polar surface area
