# Supplementary material for: Optimization of mTOR Inhibitors Using Property-Based Drug Design and Free–Wilson Analysis for Improved In Vivo Efficacy
Source: ACS Med Chem Lett. 2023 Oct 25;14(11):1544–50. doi: 10.1021/acsmedchemlett.3c00351 (PMC10641921; doi:10.1021/acsmedchemlett.3c00351)

Supplementary Information for:

Optimization of mTOR Inhibitors Using Property-Based Drug Design and Free–Wilson Analysis for Improved In Vivo Efficacy

Sean T. Murphy,* Joy Atienza, Jason W. Brown, Zacharia S. Cheruvallath, Matthew J. Cukierski, Robyn Fabrey, Walter Keung, Lily Kwok, Shawn O'Connell, Mingnam Tang, Darin L. Vanderpool, Patrick W. Vincent, Lilly Zhang, Matthew A. Marx

Takeda California, 9625 Towne Centre Drive, San Diego, CA 92121, United States

Table of Contents

1. **Synthetic procedures** **S3-S27**
2. **HPLC Chromatograms for In Vivo Compounds** **S28**
3. **Cellular Assay Counts and Standard Deviations** **S33**
4. **In vitro assays** **S34**
5. **In vivo assays** **S36**
6. **TGI Body Weight Data** **S36**

General Chemistry Experimental

Starting materials were either available commercially or prepared as described by Jin et al.[Jin-refs] All references to ether are diethyl ether; brine refers to a saturated aqueous solution of NaCl. Unless otherwise indicated, all temperatures are expressed in °C (degrees Celsius). All reactions conducted under an inert atmosphere at room temperature unless otherwise noted. ^{1}H NMR spectra were recorded on a Bruker Avance III 400. Chemical shifts are expressed in parts per million (ppm). Coupling constants are in units of Hertz (Hz). Splitting patterns describe apparent multiplicities and are designated as s (singlet), d (doublet), t (triplet), q (quartet), m (multiplet), br (broad). Low-resolution mass spectra (LRMS) and compound purity data were acquired on a Waters ACQUITY UPLC system equipped with electrospray ionization (ESI) source (calibrated to within +/- 0.2 mass units), UV diode array detector (210-400 nm), and evaporative light scattering detector (ELSD). Prep HPLC was conducted on a Waters ZQ LC/MS single quadrupole system equipped with electrospray ionization (ESI) source and UV detector (220 and 254 nm) using either acidic mode (eluted with 0.05% TFAA in water and 0.035% TFAA in acetonitrile) or basic mode (eluted with 10 mM ammonium bicarbonate in water and 80/20 (v/v) acetonitrile/10 mM aqueous ammonium bicarbonate). Thin-layer chromatography was performed on 0.25 mm E. Merck silica gel plates (60F-254), visualized with UV light, 5% ethanolic phosphomolybdic acid, Ninhydrin or p-anisaldehyde solution. Flash column chromatography was performed on ISCO CombiFlash systems using ISCO pre-packed columns. All final compounds tested were at least 95% pure by NMR and UPLC (UV diode array detector, 210-400 nm). The purity for in vivo test compounds was determined by HPLC on an Agilent 1260 Infinity II instrument. HPLC conditions were as follows: Kinetex 2.6 µm C18 100 Å LC Column 50 x 2.1 mm, 36 °C, 5−100% ACN (0.1% TFAA) in water (0.1% TFAA), 4 min run, flow rate 1.0 mL/min, UV detection (λ = 215, 254 nm). No unexpected or unusually high safety hazards were encountered.

References

Jin, S.; Mikami, S.; Scorah, N.; Chen, Y.; Halkowycz, P.; Shi, L.; Kahana, J.; Vincent, P.; Jong, R. D.; Atienza, J.; Fabrey, R.; Zhang, L.; Lardy, M. Rational Discovery of a Highly Novel and Selective MTOR Inhibitor. Bioorganic & Medicinal Chemistry Letters **2019**, 29 (21), 126659.

Jin, B.; Scorah, N.; Dong, Q. Hexahydroxinopterine Compounds. U.S. Patent 8,163,755, April 24, **2012**.

Jin, B.; Scorah, N.; Dong, Q. Hexahydroxinopterine Compounds. U.S. Patent 8,268,819, September 18, **2012**.

Hicks, F.; Hou, Y.; Langston, M.; Mccarron, A.; O'Brien, E.; Ito, T.; Ma, C.; Matthews, C.; O'Bryan, C.; Provencal, D.; Zhao, Y.; Huang, J.; Yang, Q.; Heyang, L.; Johnson, M.; Sitang, Y.; Yuqiang, L. Development of a Practical Synthesis of a TORC1/2 Inhibitor: A Scalable Application of Memory of Chirality. *Organic Process Research & Development* **2013**, 17 (5), 829–837.

Bertus, P.; Szymoniak, J. Ti(II)-Mediated Conversion of α-Heterosubstituted (O, N, S) Nitriles to Functionalized Cyclopropylamines. Effect of Chelation on the Cyclopropanation Step. *The Journal of Organic Chemistry* **2002**, 67 (11), 3965–3968.

Abbreviations

ACN, acetonitrile; DIPEA, N,N-diisopropylethylamine; dppf, 1,1'-Bis(diphenylphosphino)ferrocene; EDCI, 1-ethyl-3-(3-dimethylaminopropyl)carbodiimide; HOBT, hydroxybenzotriazole; MC, methyl cellulose; MSA, methanesulfonic acid; NaHMDS, sodium bis(trimethylsilyl)amide; NMP, N-methyl-2-pyrrolidinone; TFA salt, trifluoroacetate salt; TFAA, trifluoroacetic acid.

Cmpd 4a

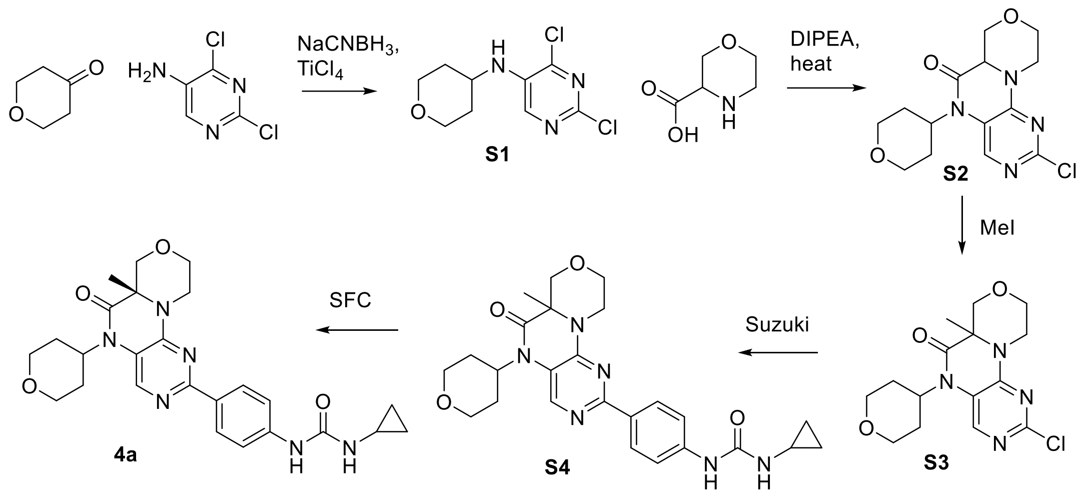

S1. To 2,4-dichloropyrimidin-5-amine (2.0 g, 12.20 mmol) in 40.0 mL of DCM was added dihydro-2H-pyran-4(3H)-one (1.463 mL, 15.85 mmol) and titanium tetrachloride (13.42 mL, 13.42 mmol). The reaction mixture was stirred at 20 °C for 3.5 h. Sodium cyanoborohydride (2.299 g, 36.6 mmol) was added in 4 equal portions over 10 min and the reaction was stirred at 20 °C for 18 h. Another 0.5 eq of titanium tetrachloride (6.1 mL of 1M solution) and reaction stirred at 20 °C for 18 h. The reaction was washed with water (150 mL) and the aqueous layer was extracted with additional DCM (3 x 30 mL). The combined organics were washed with brine, dried with sodium sulfate, and concentrated in vacuo to give 2,4-dichloro-N-(tetrahydro-2H-pyran-4-yl)pyrimidin-5-amine (2.59 g, 86%) as an orange semisolid. The crude was used without further purification. LRMS m/z: $[M+H]^+$ calcd for $C_9H_{11}Cl_2N_3O$, 248.0; found, 248.1.

S2. To 2,4-dichloro-N-(tetrahydro-2H-pyran-4-yl)pyrimidin-5-amine (2.59 g, 10.44 mmol) in 2.0 mL of DMSO was added morpholine-3-carboxylic acid hydrochloride (2.274 g, 13.57 mmol) and DIPEA (9.12 mL, 52.2 mmol) and the mixture was stirred at 100 °C for 18 h. The reaction was diluted with 150 mL of water and extracted with ethyl acetate (4 x 40 mL). The combined organics were washed with brine, dried with magnesium sulfate, and concentrated in vacuo. The crude (1.4 g) was purified by prep-HPLC (25-35% ACN, acidic mode) to give 2-chloro-5-(tetrahydro-2H-pyran-4-yl)-6a,7,9,10-tetrahydro-[1,4]oxazino[3,4-h]pteridin-6(5H)-one (0.28 g, 8.3%) as a light yellow solid. LRMS m/z: $[M+H]^+$ calcd for $C_{14}H_{17}ClN_4O_3$, 325.1; found, 325.2. ^1H NMR (400 MHz, CHLOROFORM-d) δ ppm 1.34 - 1.85 (m, 4 H) 2.48 - 2.77 (m, 2 H) 2.96 - 3.23 (m, 1 H) 3.38 - 3.67 (m, 4 H) 3.95 - 4.26 (m, 2 H) 4.36 - 4.72 (m, 3 H) 8.10 (s, 1 H).

S3. A solution of 2-chloro-5-(tetrahydro-2H-pyran-4-yl)-6a,7,9,10-tetrahydro-[1,4]oxazino[3,4-h]pteridin-6(5H)-one (0.200 g, 0.616 mmol) in DMSO (1.2 mL) was frozen in a dry ice-acetone bath. Sodium tert-butoxide (0.065 g, 0.677 mmol) was added followed by DMSO (1.0 mL) and then iodomethane (0.042 mL, 0.677 mmol). The cooling bath was removed, and the mixture was allowed to warm up to 20 °C and stir for 12 h. More iodomethane (0.154 mmol, 0.022 g, 0.0096 mL) was added and the reaction stirred for 18 h. The reaction was diluted with 30 mL of water and extracted with ethyl acetate (3 x 15 mL). The combined organics were washed with brine, dried with magnesium sulfate, and concentrated in vacuo to give 2-chloro-6a-methyl-5-(tetrahydro-2H-pyran-4-yl)-6a,7,9,10-tetrahydro-[1,4]oxazino[3,4-

h]pteridin-6(5H)-one (0.164 g, 79%) as a brown foam which was used without further purification. LRMS m/z: $[M+H]^+$ calcd for $C_{15}H_{19}ClN_4O_3$, 339.1; found, 339.3.

S4. A mixture of 2-chloro-6a-methyl-5-(tetrahydro-2H-pyran-4-yl)-6a,7,9,10-tetrahydro-[1,4]oxazino[3,4-h]pteridin-6(5H)-one (0.140 g, 0.413 mmol), 1-cyclopropyl-3-(4-(4,4,5,5-tetramethyl-1,3,2-dioxaborolan-2-yl)phenyl)urea (0.137 g, 0.455 mmol), $PdCl_2$(dppf) (0.060 g, 0.083 mmol) and saturated aqueous sodium bicarbonate (1.050 mL, 0.413 mmol) in 1,4-dioxane (4.13 mL) was heated by microwave irradiation at 100 °C for 1 h. More $PdCl_2$(dppf) (0.060 g, 0.083 mmol) was added, and the mixture heated in the microwave at 100 °C for 2 h. More $PdCl_2$(dppf) (0.060 g, 0.083 mmol) was added and heated in the microwave at 120°C for 1h. The reaction was filtered to remove solids and purified by prep HPLC (23-30% ACN, acidic mode) to give racemic (±)-1-cyclopropyl-3-(4-(6a-methyl-6-oxo-5-(tetrahydro-2H-pyran-4-yl)-5,6,6a,7,9,10-hexahydro-[1,4]oxazino[3,4-h]pteridin-2-yl)phenyl)urea TFA salt (64 mg, 32%) as a white solid. LRMS m/z: $[M+H]^+$ calcd for $C_{25}H_{30}N_6O_4$, 479.2; found, 479.4. ^{1}H NMR (400 MHz, DMSO-d6) δ ppm 0.36 - 0.46 (m, 2 H) 0.59 - 0.69 (m, 2 H) 1.30 (s, 3 H) 1.55 - 1.73 (m, 2 H) 2.41 - 2.48 (m, 1 H) 2.52 - 2.70 (m, 2 H) 3.24 (td, J=13.14, 4.04 Hz, 1 H) 3.33 - 3.45 (m, 1 H) 3.47 - 3.73 (m, 3 H) 3.86 - 4.22 (m, 5 H) 4.34 - 4.50 (m, 1 H) 6.50 (br. s., 1 H) 7.52 (d, J=8.8 Hz, 2 H) 8.18 (d, J=8.8 Hz, 2 H) 8.51 (s, 1 H) 8.62 (s, 1 H).

4a. Racemic (±)-1-cyclopropyl-3-(4-(6a-methyl-6-oxo-5-(tetrahydro-2H-pyran-4-yl)-5,6,6a,7,9,10-hexahydro-[1,4]oxazino[3,4-h]pteridin-2-yl)phenyl)urea (59 mg) was separated by chiral SFC using a Chiralpak AD-H column eluted with CO_2 and isopropanol to give (S)-1-cyclopropyl-3-(4-(6a-methyl-6-oxo-5-(tetrahydro-2H-pyran-4-yl)-5,6,6a,7,9,10-hexahydro-[1,4]oxazino[3,4-h]pteridin-2-yl)phenyl)urea (12 mg, 20% yield, 1st eluting fraction) as a white solid. LRMS m/z: $[M+H]^+$ calcd for $C_{25}H_{30}N_6O_4$, 479.2; found, 479.4. ^{1}H NMR (400 MHz, DMSO-d6) δ ppm 0.37 - 0.45 (m, 2 H) 0.60 - 0.69 (m, 2 H) 1.25 (s, 3 H) 1.54 - 1.74 (m, 2 H) 2.39 - 2.72 (m, 3 H) 3.21 (td, J=13.01, 3.79 Hz, 1 H) 3.35 - 3.46 (m, 1 H) 3.48 - 3.70 (m, 3 H) 3.86 - 4.01 (m, 3 H) 4.01 - 4.13 (m, 2 H) 4.35 - 4.51 (m, 1 H) 6.46 (s, 1 H) 7.44 - 7.55 (m, 2 H) 8.19 (d, J=8.6 Hz, 2 H) 8.54 (d, J=8.8 Hz, 2 H).

Cmpd 3b

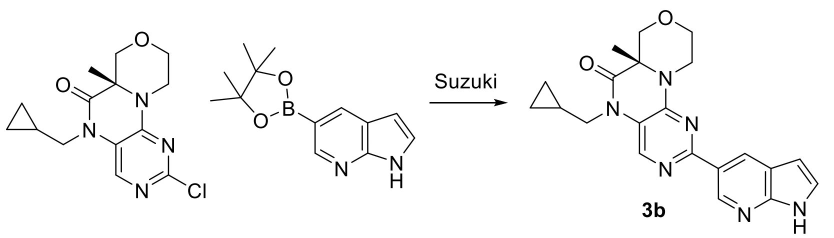

3b. A mixture of (S)-2-chloro-5-(cyclopropylmethyl)-6a-methyl-6a,7,9,10-tetrahydro-[1,4]oxazino[3,4-h]pteridin-6(5H)-one (0.157 g, 0.508 mmol), 5-(4,4,5,5-tetramethyl-1,3,2-dioxaborolan-2-yl)-1H-pyrrolo[2,3-b]pyridine (0.248 g, 1.017 mmol) and [1,1'-bis(diphenylphosphino)ferrocene]-dichloropalladium(II) (0.019 g, 0.025 mmol) in a mixture of 1,4-dioxane (12.56 mL) and aqueous saturated sodium bicarbonate solution (6.28 mL) was heated by microwave at 140 °C for 30 m. The reaction was diluted with ethyl acetate (50 mL), washed with water (50 mL), dried with magnesium sulfate, and concentrated in vacuo. The crude material was purified by silica gel chromatography eluting

with 0 - 5% of methanol:DCM to give (S)-5-(cyclopropylmethyl)-6a-methyl-2-(1H-pyrrolo[2,3-b]pyridin-5-yl)-6a,7,9,10-tetrahydro-[1,4]oxazino[3,4-h]pteridin-6(5H)-one (112 mg, 0.287 mmol, 56.4 % yield) as an off-white solid. LRMS m/z: $[M+H]^+$ calcd for $C_{21}H_{22}N_6O_2$, 391.2; found, 391.2. ^1H NMR (400 MHz, DMSO-d6) δ ppm 0.31 - 0.55 (m, 4 H) 1.20 (s, 1 H) 1.38 (s, 3 H) 3.32 (br. s., 1 H) 3.57 - 3.66 (m, 1 H) 3.70 (d, J=12.13 Hz, 1 H) 3.83 (dd, J=14.40, 7.33 Hz, 1 H) 3.91 - 4.04 (m, 2 H) 4.06 - 4.13 (m, 1 H) 4.27 (d, J=11.12 Hz, 1 H) 6.57 (dd, J=3.28, 1.77 Hz, 1 H) 7.50 - 7.55 (m, 1 H) 8.43 (s, 1 H) 8.84 (d, J=1.26 Hz, 1 H) 9.21 (d, J=2.02 Hz, 1 H) 11.83 (s, 1 H).

Cmpd 4b

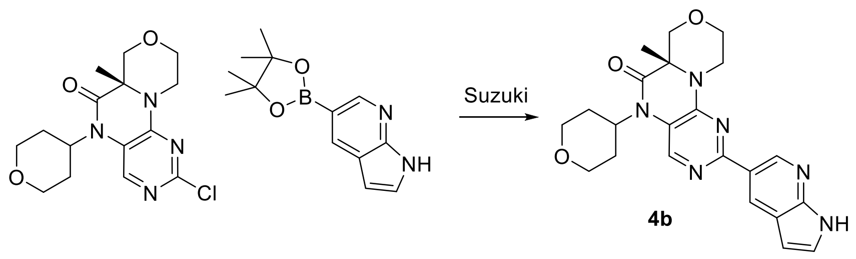

4b. A mixture of (S)-2-chloro-6a-methyl-5-(tetrahydro-2H-pyran-4-yl)-6a,7,9,10-tetrahydro-[1,4]oxazino[3,4-h]pteridin-6(5H)-one (150 mg, 0.443 mmol), 5-(4,4,5,5-tetramethyl-1,3,2-dioxaborolan-2-yl)-1H-pyrrolo[2,3-b]pyridine (140 mg, 0.576 mmol) , $PdCl_2$(dppf)-DCM adduct (18.08 mg, 0.022 mmol) and tribasic potassium phosphate (235 mg, 1.107 mmol) in 1,4-dioxane (1.5 mL) and water (0.75 mL) was heated in the microwave at 110 °C for 1 h. The resulting slurry was stirred with water (2 mL) and ethyl acetate (2 mL) for 15 min. The solid was collected by vacuum filtration and rinsed with ethyl acetate to give (S)-6a-methyl-2-(1H-pyrrolo[2,3-b]pyridin-5-yl)-5-(tetrahydro-2H-pyran-4-yl)-6a,7,9,10-tetrahydro-[1,4]oxazino[3,4-h]pteridin-6(5H)-one (165 mg, 0.392 mmol, 89 % yield) as a gray solid. LRMS m/z: $[M+H]^+$ calcd for $C_{22}H_{24}N_6O_3$, 421.2; found, 421.4. ^1H NMR (400 MHz, DMSO-d6) δ ppm 1.28 (s, 3 H) 1.61 (d, J=10.9 Hz, 1 H) 1.70 (d, J=12.1 Hz, 1 H) 2.42 - 2.48 (m, 1 H) 2.58 - 2.74 (m, 1 H) 3.26 (td, J=13.1, 4.0 Hz, 1 H) 3.40 (t, J=10.9 Hz, 1 H) 3.48 - 3.71 (m, 3 H) 3.87 - 4.01 (m, 3 H) 4.08 (dd, J=11.2, 3.7 Hz, 1 H) 4.16 (dd, J=13.6, 2.3 Hz, 1 H) 4.45 (t, J=11.9 Hz, 1 H) 6.57 (d, J=3.3 Hz, 1 H) 7.52 (d, J=2.0 Hz, 1 H) 8.58 (s, 1 H) 8.82 (d, J=2.0 Hz, 1 H) 9.20 (d, J=2.0 Hz, 1 H) 11.83 (s, 1 H)

Cmpd 3c

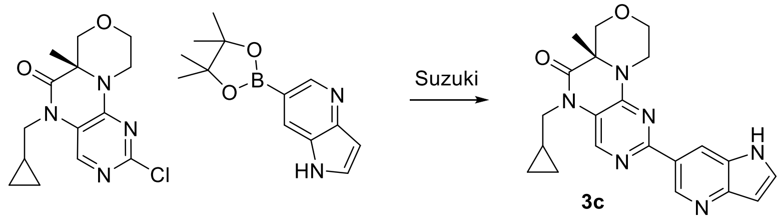

3c. A mixture of (S)-2-chloro-5-(cyclopropylmethyl)-6a-methyl-6a,7,9,10-tetrahydro-[1,4]oxazino[3,4-h]pteridin-6(5H)-one (50 mg, 0.162 mmol), 6-(4,4,5,5-tetramethyl-1,3,2-dioxaborolan-2-yl)-1H-pyrrolo[3,2-b]pyridine (79 mg, 0.324 mmol), and $PdCl_2$(dppf)-DCM adduct (5.92 mg, 8.10 μmol) in 1,4-

dioxane (6 mL) and saturated aqueous sodium bicarbonate (3.0 mL) was heated at 140 °C for 30 min in the microwave. The mixture was filtered, and the filtrate was concentrated in vacuo. The residue was purified by prep HPLC (acidic mode) to give (S)-5-(cyclopropylmethyl)-6a-methyl-2-(1H-pyrrolo[3,2-b]pyridin-6-yl)-6a,7,9,10-tetrahydro-[1,4]oxazino[3,4-h]pteridin-6(5H)-one TFA salt (37 mg, 45%) as a light yellow solid. LRMS m/z: $[M+H]^+$ calcd for $C_{21}H_{22}N_6O_2$, 391.2; found, 391.3. ^1H NMR (400 MHz, METHANOL-d4) δ ppm 0.57 (dd, J=5.05, 2.27 Hz, 4 H) 1.17 - 1.32 (m, 1 H) 1.50 (s, 3 H) 3.36 - 3.50 (m, 1 H) 3.77 (d, J=11.87 Hz, 2 H) 3.90 (d, J=6.82 Hz, 1 H) 4.00 (d, J=7.07 Hz, 1 H) 4.13 (d, J=11.62 Hz, 2 H) 4.29 - 4.47 (m, 1 H) 6.92 (dd, J=3.16, 0.88 Hz, 1 H) 8.20 (d, J=3.28 Hz, 1 H) 8.38 (s, 1 H) 9.31 - 9.42 (m, 1 H) 9.45 (d, J=1.26 Hz, 1 H).

Cmpd 4c

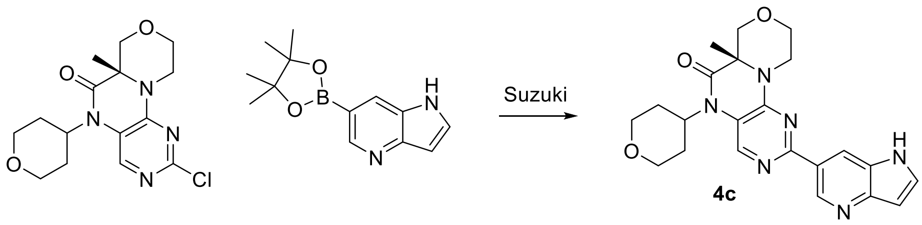

4c. A mixture of (S)-2-chloro-6a-methyl-5-(tetrahydro-2H-pyran-4-yl)-6a,7,9,10-tetrahydro-[1,4]oxazino[3,4-h]pteridin-6(5H)-one (50 mg, 0.148 mmol), 6-(4,4,5,5-tetramethyl-1,3,2-dioxaborolan-2-yl)-1H-pyrrolo[3,2-b]pyridine (64.8 mg, 0.266 mmol), $PdCl_2$(dppf)-DCM adduct (6.03 mg, 7.38 µmol), and tribasic potassium phosphate(78 mg, 0.369 mmol) in 1,4-dioxane (520 µL) and water (260 µL) was heated in a microwave for 30 min at 110 °C. The crude reaction was diluted with ethyl acetate (4.0 mL) and partitioned with 1M aqueous monobasic potassium phosphate pH=4.6 (2.0 mL). The layers were separated, and the organic phase was washed with brine (2 mL), dried over sodium sulfate, filtered, and concentrated in vacuo. The resulting crude material was reconstituted in DMSO (2.0 mL), filtered, rinsed with DMSO (2 x 0.5 mL) and purified via preparative HPLC using a gradient eluant of 15-40% ACN:0.05% aqueous trifluoroacetic acid to provide the TFA salt of the product. The TFA salt was dissolved in methanol (2 mL) and passed through a 200 mg StratoSpheres SPE cartridge (PL-HCO3 MP SPE) to remove TFAA. The cartridge was rinsed with methanol (3 x 1 mL) and the filtrate was dried in vacuo to provide (S)-6a-methyl-2-(1H-pyrrolo[3,2-b]pyridin-6-yl)-5-(tetrahydro-2H-pyran-4-yl)-6a,7,9,10-tetrahydro-[1,4]oxazino[3,4-h]pteridin-6(5H)-one (34.5 mg, 0.082 mmol, 55.6 % yield) as an off-white solid. LRMS m/z: $[M+H]^+$ calcd for $C_{22}H_{24}N_6O_3$, 421.2; found, 421.3. ^1H NMR (400 MHz, DMSO-d6) δ ppm 1.29 (s, 3 H) 1.57 - 1.66 (m, 1 H) 1.66 - 1.76 (m, 1 H) 2.42 - 2.49 (m, 1 H) 2.66 (qd, J=12.2, 4.7 Hz, 1 H) 3.22 - 3.32 (m, 1 H) 3.36 - 3.45 (m, 1 H) 3.50 - 3.58 (m, 1 H) 3.58 - 3.65 (m, 1 H) 3.67 (d, J=11.6 Hz, 1 H) 3.87 - 4.01 (m, 3 H) 4.04 - 4.19 (m, 2 H) 4.39 - 4.53 (m, 1 H) 6.61 (d, J=2.5 Hz, 1 H) 7.76 (d, J=2.8 Hz, 1 H) 8.60 (s, 1 H) 8.61 (s, 1 H) 9.31 (d, J=1.8 Hz, 1 H) 11.49 (br. s., 1 H).

Cmpd 4d

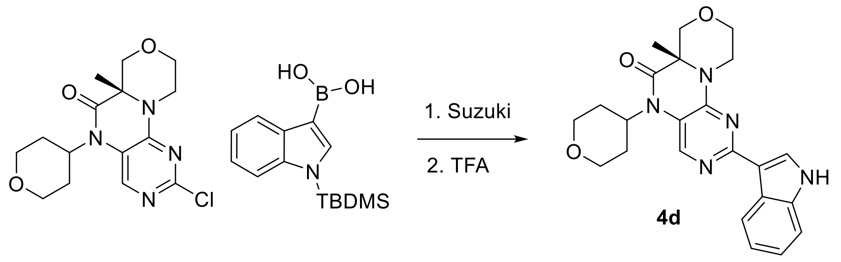

4d. A mixture of (S)-2-chloro-6a-methyl-5-(tetrahydro-2H-pyran-4-yl)-6a,7,9,10-tetrahydro-[1,4]oxazino[3,4-h]pteridin-6(5H)-one (70 mg, 0.207 mmol), 1-(tert-butyldimethylsilyl)-1H-indol-3-ylboronic acid (114 mg, 0.413 mmol) and [1,1'-bis(diphenylphosphino)ferrocene]-dichloropalladium(II) (7.56 mg, 10.33 µmol) in 1,4-dioxane (7.65 mL) and saturated aqueous sodium bicarbonate (3.83 mL) was heated at 140 °C for 30 m. The mixture was concentrated in vacuo, slurried in DCM and filtered through Celite. The filtrate was concentrated in vacuo to give 110 mg of crude (S)-2-(1-(tert-butyldimethylsilyl)-1H-indol-3-yl)-6a-methyl-5-(tetrahydro-2H-pyran-4-yl)-6a,7,9,10-tetrahydro-[1,4]oxazino[3,4-h]pteridin-6(5H)-one which was used without further purification. To a solution of (S)-2-(1-(tert-butyldimethylsilyl)-1H-indol-3-yl)-6a-methyl-5-(tetrahydro-2H-pyran-4-yl)-6a,7,9,10-tetrahydro-[1,4]oxazino[3,4-h]pteridin-6(5H)-one (110 mg, 0.206 mmol) in 8 mL of DCM was added 2 mL of TFAA and the reaction stirred at 20 °C for 30 min. The solution was concentrated to dryness and the residue was dissolved in DMF (2 mL), filtered, and purified by prep HPLC (acidic mode) to give (S)-2-(1H-indol-3-yl)-6a-methyl-5-(tetrahydro-2H-pyran-4-yl)-6a,7,9,10-tetrahydro-[1,4]oxazino[3,4-h]pteridin-6(5H)-one TFA salt (10 mg, 0.024 mmol, 9%) as a tan solid. LRMS m/z: $[M+H]^+$ calcd for $C_{23}H_{25}N_5O_3$, 420.2; found, 420.3. ^1H NMR (400 MHz, DMSO-d6) δ ppm 1.32 (br. s., 3 H) 1.56 - 1.76 (m, 4 H) 3.33 - 3.45 (m, 2 H) 3.50 - 3.74 (m, 4 H) 3.90 - 4.00 (m, 2 H) 4.11 (dd, J=11.2, 1.1 Hz, 1 H) 4.41 (t, J=12.6 Hz, 1 H) 6.39 - 6.64 (m, 1 H) 7.08 - 7.26 (m, 2 H) 7.47 (d, J=7.1 Hz, 1 H) 8.18 (br. s., 1 H) 8.36 - 8.50 (m, 2 H) 11.69 (s, 1 H).

Cmpd 4e

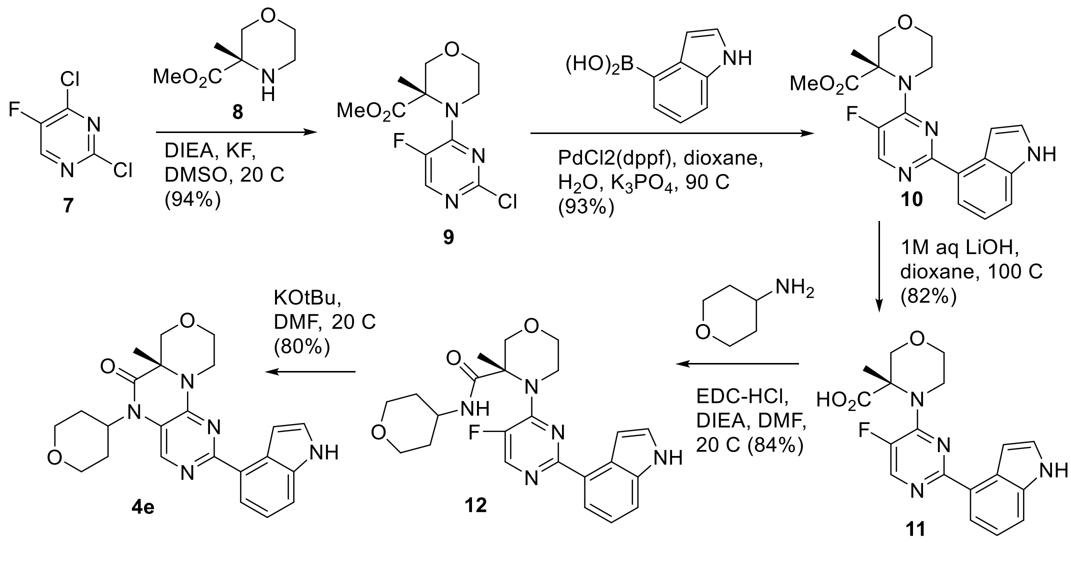

9. A 3-neck, round bottom, reaction flask equipped with a mechanical stirrer, an addition funnel, and a nitrogen inlet was charged with (*S*)-3-(methoxycarbonyl)-3-methylmorpholin-4-ium (7,7-dimethyl-2-oxobicyclo[2.2.1]heptan-1-yl)methanesulfonate (148.7 g, 380 mmol), 2,4-dichloro-5-fluoropyrimidine (57.7 g, 345 mmol) and DMSO (691 mL) to give a white suspension. Next, potassium fluoride (26.1 g, 449 mmol) was added at 23°C followed by DIPEA (150 mL, 863 mmol) from the addition funnel over a 10 min period. The mixture was stirred at 23 °C for 3 days under nitrogen to give a yellow-orange suspension. The reaction mixture cooled on ice to 12 °C and diluted with saturated aqueous ammonium chloride (1.4 L), which was added dropwise over a 2 h period. A gum formed on the stir bar. The gum was taken up in ethyl acetate, washed with brine and combined with the extracts from the aqueous phase. The aqueous phase was extracted with ethyl acetate (750 mL), washed with brine (750 mL), dried over magnesium sulfate, filtered, rinsed with ethyl acetate, and concentrated in vacuo. The residue was taken up in diethyl ether (150 mL) and concentrated in vacuo. This was repeated three times to provide methyl (S)-4-(2-chloro-5-fluoropyrimidin-4-yl)-3-methylmorpholine-3-carboxylate (93.6 g, 94%) as a yellow-orange solid. LRMS m/z: $[M+H]^+$ calcd for $C_{11}H_{13}ClFN_3O_3$, 290.1; found, 290.1. ^1H NMR (400 MHz, DMSO-d_6) δ ppm 1.55 (s, 3 H), 3.51 - 3.59 (m, 1 H), 3.61 (s, 3 H), 3.68 - 3.81 (m, 3 H), 3.90 - 4.02 (m, 2 H), 8.38 (d, *J*=6.1 Hz, 1 H).

10. A 2 L round-bottomed flask equipped with a reflux condenser and a magnetic stir bar was charged with (*S*)-methyl 4-(2-chloro-5-fluoropyrimidin-4-yl)-3-methylmorpholine-3-carboxylate (93 g, 321 mmol), 4-(4,4,5,5-tetramethyl-1,3,2-dioxaborolan-2-yl)-1*H*-indole (82 g, 337 mmol), $PdCl_2$(dppf) (7.05 g, 9.63 mmol), and potassium phosphate (76 g, 803 mmol) in 1,4-dioxane (535 mL) and water (535 mL). The reaction mixture was stirred at 90 °C for 18 h and then cooled to 23 °C. The contents of the flask were filtered through Celite and the Celite was rinsed with ethyl acetate, which was combined with the filtrate. The filtrate was further diluted with ethyl acetate, washed with saturated aqueous sodium bicarbonate, saturated aqueous ammonium chloride, and brine, and then dried over magnesium sulfate. The product-containing mixture was filtered, and the solvent removed in vacuo. The residue was treated with diethyl ether (200 mL) and concentrated in vacuo. The diethyl ether wash was repeated three times and concentrated in vacuo to give methyl (S)-4-(5-fluoro-2-(1H-indol-4-yl)pyrimidin-4-yl)-3-methylmorpholine-3-carboxylate as a brown solid (119 g, 100%). LRMS m/z: $[M+H]^+$ calcd for $C_{19}H_{19}FN_4O_3$, 371.1; found, 371.3. ^1H NMR (400 MHz, DMSO-d_6) δ ppm 1.70 (s, 3 H), 3.51 (s, 3 H), 3.54 - 3.63 (m, 1 H), 3.70 - 3.76 (m, 1 H), 3.78 - 3.88 (m, 2 H), 3.91 - 4.02 (m, 2 H), 7.19 (t, *J*=7.71 Hz, 1 H), 7.37 (d, *J*=2.02 Hz, 1 H), 7.44 (t, *J*=2.78 Hz, 1 H), 7.54 (d, *J*=7.83 Hz, 1 H), 7.86 (d, *J*=7.07 Hz, 1 H), 8.60 (d, *J*=5.81 Hz, 1 H), 11.28 (br s, 1 H).

11. A solution of (S)-methyl 4-(5-fluoro-2-(1H-indol-4-yl)pyrimidin-4-yl)-3-methylmorpholine-3-carboxylate (5.6 g, 15.12 mmol) in 1,4-dioxane (80 ml) and 1.0 M aqueous lithium hydroxide solution (80 ml, 80 mmol) was heated at 100 °C for 10 h. The solution was concentrated in vacuo to remove 1,4-dioxane and then diluted with water (50 mL) and acidified with 1 M aqueous hydrochloric acid (80 mL) to pH 4 (a yellow solid formed as the solution was acidified). The solid was collected by vacuum filtration, rinsed with water, and dried under vacuum to give (S)-4-(5-fluoro-2-(1H-indol-4-yl)pyrimidin-4-yl)-3-methylmorpholine-3-carboxylic acid (4.426 g, 12.42 mmol, 82 % yield) as a light yellow solid. LRMS m/z: $[M+H]^+$ calcd for $C_{18}H_{17}FN_4O_3$, 357.1; found, 357.2. ^1H NMR (400 MHz, DMSO-d_6) δ ppm 1.66 (s, 3 H), 3.49 - 3.61 (m, 1 H), 3.69 (d, *J*=11.12 Hz, 1 H), 3.77 - 3.88 (m, 2 H), 3.88 - 4.00 (m, 2 H), 7.15 (t, *J*=7.71 Hz, 1 H), 7.36 (br s, 1 H), 7.42 (t, *J*=2.65 Hz, 1 H), 7.52 (d, *J*=8.08 Hz, 1 H), 7.95 (d, *J*=7.33 Hz, 1 H), 8.57 (d, *J*=6.06 Hz, 1 H), 11.24 (br s, 1 H), 12.67 (br s, 1 H).

12. To a solution of (S)-4-(5-fluoro-2-(1H-indol-4-yl)pyrimidin-4-yl)-3-methylmorpholine-3-carboxylic acid (2.500 g, 7.02 mmol), 4-aminotetrahydropyran (1.475 mL, 14.03 mmol), 1-(3-dimethylaminopropyl)-3-ethylcarbodiimide hydrochloride (2.017 g, 10.52 mmol), and HOBT (1.612 g, 10.52 mmol) in DMF (17.54 mL) was added DIPEA (3.67 mL, 21.05 mmol). The reaction mixture was stirred at 20 °C for 16 h. The reaction mixture was poured into 150 mL water and stirred for 5 m. The resulting tan precipitate was collected by vacuum filtration and rinsed with water (2 x 10 mL) to provide 1.5 g of product as a tan solid. The aqueous filtrate was extracted with ethyl acetate (3 x 30 mL). The combined organic layers were concentrated in vacuo to give 1.1 g of crude product. The combined solids were purified by NH silica gel chromatography eluted with 0 - 10% of methanol/DCM to give (S)-4-(5-fluoro-2-(1H-indol-4-yl)pyrimidin-4-yl)-3-methyl-N-(tetrahydro-2H-pyran-4-yl)morpholine-3-carboxamide (2.6 g, 84%) as an off-white solid. LRMS m/z: $[M+H]^+$ calcd for $C_{23}H_{26}FN_5O_3$, 440.2; found, 440.4. ^1H NMR (400 MHz, DMSO-d6) δ ppm 0.96 - 1.11 (m, 1 H) 1.11 - 1.21 (m, 1 H) 1.41 - 1.55 (m, 2 H) 1.59 - 1.73 (m, 3 H) 3.01 (td, J=11.5, 2.3 Hz, 1 H) 3.17 - 3.26 (m, 1 H) 3.38 - 3.54 (m, 2 H) 3.60 (d, J=11.1 Hz, 1 H) 3.66 - 3.81 (m, 3 H) 3.81 - 4.07 (m, 3 H) 7.03 - 7.16 (m, 1 H) 7.31 - 7.42 (m, 2 H) 7.49 (d, J=7.8 Hz, 1 H) 7.52 - 7.67 (m, 1 H) 7.95 - 8.07 (m, 1 H) 8.50 - 8.63 (m, 1 H) 11.22 (br. s., 1 H).

4e. To a solution of (S)-4-(5-fluoro-2-(1H-indol-4-yl)pyrimidin-4-yl)-3-methyl-N-(tetrahydro-2H-pyran-4-yl)morpholine-3-carboxamide (2.5 g, 5.69 mmol) in DMF (21.88 mL) was added potassium tert-butoxide (1.0 M in THF) (17.07 mL, 17.07 mmol) at 40 °C and the reaction was stirred at 40 °C for 30 m. The heating batch was removed, and the reaction was quenched with glacial acetic acid (0.814 mL, 14.22 mmol) and then water (1.1 mL). The reaction mixture was poured into 180 mL of water with stirring. The resulting precipitate was collected by vacuum filtration, rinsed with water (3 x 50 mL), and dried under high vacuum at 40 °C to give (S)-2-(1H-indol-4-yl)-6a-methyl-5-(tetrahydro-2H-pyran-4-yl)-6a,7,9,10-tetrahydro-[1,4]oxazino[3,4-h]pteridin-6(5H)-one (1.9 g, 80 %) as an off-white solid. LRMS m/z: $[M+H]^+$ calcd for $C_{23}H_{25}N_5O_3$, 420.2; found, 420.4. ^1H NMR (400 MHz, DMSO-d6) δ ppm 1.29 (s, 3 H) 1.63 (d, J=11.6 Hz, 1 H) 1.68 - 1.80 (m, 1 H) 2.42 - 2.57 (m, 1 H) 2.61 - 2.81 (m, 1 H) 3.26 - 3.35 (m, 1 H) 3.37 - 3.47 (m, 1 H) 3.52 - 3.72 (m, 3 H) 3.88 - 4.00 (m, 3 H) 4.00 - 4.17 (m, 2 H) 4.37 - 4.57 (m, 1 H) 7.19 (t, J=7.7 Hz, 1 H) 7.40 (t, J=2.1 Hz, 1 H) 7.45 (t, J=2.6 Hz, 1 H) 7.52 (d, J=8.1 Hz, 1 H) 8.11 (dd, J=7.4, 0.9 Hz, 1 H) 8.66 (s, 1 H) 11.26 (br. s., 1 H).

Cmpd 4f

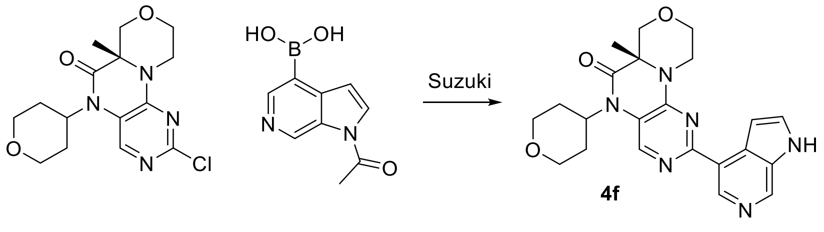

4f. A mixture of (S)-2-chloro-6a-methyl-5-(tetrahydro-2H-pyran-4-yl)-6a,7,9,10-tetrahydro-[1,4]oxazino[3,4-h]pteridin-6(5H)-one (35 mg, 0.103 mmol), 1-acetyl-1H-pyrrolo[2,3-c]pyridin-4-ylboronic acid (31.6 mg, 0.155 mmol), $PdCl_2$(dppf)-DCM adduct(4.22 mg, 5.17 µmol), and tribasic potassium phosphate (54.8 mg, 0.258 mmol) in 1,4-dioxane (360 µL) and water (180 µL) was heated in a microwave for 30 min at 110 °C. The crude reaction was concentrated via rotary evaporation. The

resulting crude material was reconstituted in DMSO (1.0 mL), filtered, rinsed with DMSO (2 x 0.25 mL), and purified by prep HPLC (acidic mode). The collected fractions were combined and concentrated via rotary evaporation to 1/4 volume. The concentrate was made basic to pH 12 with 1 M aqueous sodium hydroxide (1.5 mL) to cleave the residual N-acetyl group. The mixture was then neutralized with 1 M aqueous hydrochloric acid (1.5 mL) and concentrated via rotary evaporation to provide a mixture of salts. The salts were dissolved in methanol (15 mL) and passed through a 100 mg StratoSpheres SPE cartridge (PL-HCO3 MP SPE) to remove salt. The cartridge was rinsed with methanol (3 x 3 mL) and the filtrate was dried in vacuo to provide a solid. The solid was re-suspended in water (2 mL) and stirred for 5 min at 23 °C to furnish a white suspension which was filtered, rinsed with water and dried in vacuo to provide (S)-6a-methyl-2-(1H-pyrrolo[2,3-c]pyridin-4-yl)-5-(tetrahydro-2H-pyran-4-yl)-6a,7,9,10-tetrahydro-[1,4]oxazino[3,4-h]pteridin-6(5H)-one (13.7 mg, 0.033 mmol, 31.5 % yield) as a white solid. LRMS m/z: $[M+H]^+$ calcd for $C_{22}H_{24}N_6O_3$, 421.2; found, 421.3. ^1H NMR (400 MHz, DMSO-d6) δ ppm 1.29 (s, 3 H) 1.58 - 1.67 (m, 1 H) 1.69 - 1.79 (m, 1 H) 2.44 - 2.49 (m, 1 H) 2.62 - 2.75 (m, 1 H) 3.27 - 3.33 (m, 1 H) 3.37 - 3.46 (m, 1 H) 3.51 - 3.72 (m, 3 H) 3.89 - 4.02 (m, 3 H) 4.06 - 4.16 (m, 2 H) 4.48 (tt, J=11.91, 3.76 Hz, 1 H) 7.31 - 7.37 (m, 1 H) 7.72 (t, J=2.78 Hz, 1 H) 8.68 (s, 1 H) 8.81 (s, 1 H) 9.11 (s, 1 H) 11.78 (br. s., 1 H).

Cmpd 4g

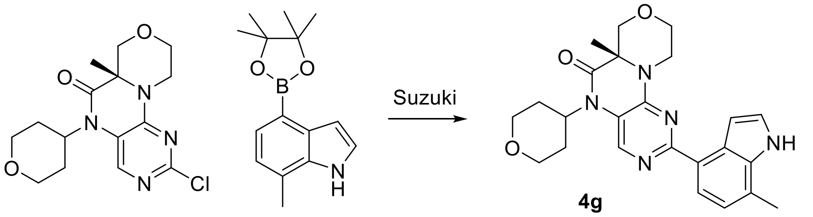

4g. Into a 20 mL microwave vial, (S)-2-chloro-6a-methyl-5-(tetrahydro-2H-pyran-4-yl)-6a,7,9,10-tetrahydro-[1,4]oxazino[3,4-h]pteridin-6(5H)-one (80 mg, 0.236 mmol), 7-methyl-4-(4,4,5,5-tetramethyl-1,3,2-dioxaborolan-2-yl)-1H-indole (121 mg, 0.472 mmol), [1,1'-bis(diphenylphosphino)ferrocene]-dichloropalladium(II) (8.64 mg, 0.012 mmol) were combined in a mixture of 1,4-dioxane (8.75 mL) and saturated aqueous sodium bicarbonate (4.4 mL). The resulting mixture was heated at 140°C for 30 m. The crude mixture was filtered through Celite, concentrated to dryness then dissolved in DMF (2 mL) and filtered through a PTFE filter. The filtrate was purified by prep HPLC (acidic mode) to give (S)-6a-methyl-2-(7-methyl-1H-indol-4-yl)-5-(tetrahydro-2H-pyran-4-yl)-6a,7,9,10-tetrahydro-[1,4]oxazino[3,4-h]pteridin-6(5H)-one TFA salt (91 mg, 70%) as a yellow solid. LRMS m/z: $[M+H]^+$ calcd for $C_{24}H_{27}N_5O_3$, 434.2; found, 434.4. ^1H NMR (400 MHz, DMSO-d6) δ ppm 1.34 (s, 3 H) 1.63 (d, J=14.1 Hz, 1 H) 1.72 (d, J=10.6 Hz, 1 H) 2.54 (s, 3 H) 2.61 - 2.73 (m, 1 H) 3.27 - 3.45 (m, 1 H) 3.45 - 3.77 (m, 4 H) 3.89 - 4.06 (m, 4 H) 4.06 - 4.25 (m, 2 H) 7.03 (d, J=7.8 Hz, 1 H) 7.35 (br. s., 1 H) 7.47 (t, J=2.8 Hz, 1 H) 7.95 (d, J=7.6 Hz, 1 H) 8.59 (s, 1 H) 11.31 (br. s., 1 H).

Cmpd 4h

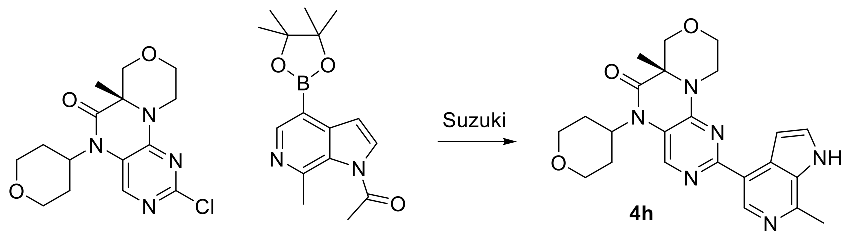

4h. A mixture of (S)-2-chloro-6a-methyl-5-(tetrahydro-2H-pyran-4-yl)-6a,7,9,10-tetrahydro-[1,4]oxazino[3,4-h]pteridin-6(5H)-one (80 mg, 0.236 mmol), 1-(7-methyl-4-(4,4,5,5-tetramethyl-1,3,2-dioxaborolan-2-yl)-1H-pyrrolo[2,3-c]pyridin-1-yl)ethanone (142 mg, 0.472 mmol), and [1,1'-bis(diphenylphosphino)ferrocene]-dichloropalladium(II) (8.64 mg, 0.012 mmol) were combined in a mixture of 1,4-dioxane (8.75 mL) and saturated aqueous sodium bicarbonate (4.4 mL) and heated at 140 °C for 30 min in the microwave. The reaction mixture was filtered through Celite, concentrated to dryness, dissolved in DMF (2 mL) and filtered. The crude solution was purified by prep HPLC (acidic mode) to give (S)-6a-methyl-2-(7-methyl-1H-pyrrolo[2,3-c]pyridin-4-yl)-5-(tetrahydro-2H-pyran-4-yl)-6a,7,9,10-tetrahydro-[1,4]oxazino[3,4-h]pteridin-6(5H)-one TFA salt (40 mg, 0.092 mmol, 39 % yield) as a beige solid. LRMS m/z: $[M+H]^+$ calcd for $C_{23}H_{26}N_6O_3$, 435.2; found, 435.4. ^1H NMR (400 MHz, DMSO-d6) δ ppm 1.32 (s, 3 H) 1.63 (d, J=11.9 Hz, 1 H) 1.73 (d, J=15.4 Hz, 1 H) 2.42 - 2.57 (m, 1 H) 2.63 - 2.72 (m, 1 H) 3.01 (s, 3 H) 3.28 - 3.48 (m, 2 H) 3.48 - 3.76 (m, 3 H) 3.90 - 4.07 (m, 3 H) 4.07 - 4.19 (m, 2 H) 4.50 (t, J=11.7 Hz, 1 H) 7.66 - 7.77 (m, 1 H) 8.40 (t, J=2.9 Hz, 1 H) 8.72 (s, 1 H) 8.90 (s, 1 H) 13.30 (br. s., 1 H).

Cmpd 4i

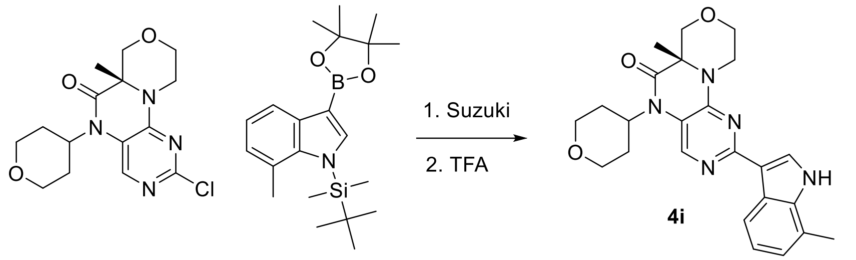

4i. A mixture of (S)-2-chloro-6a-methyl-5-(tetrahydro-2H-pyran-4-yl)-6a,7,9,10-tetrahydro-[1,4]oxazino[3,4-h]pteridin-6(5H)-one (0.1 g, 0.295 mmol), 1-(tert-butyldimethylsilyl)-7-methyl-3-(4,4,5,5-tetramethyl-1,3,2-dioxaborolan-2-yl)-1H-indole (0.110 g, 0.295 mmol), and [1,1'-bis(diphenylphosphino)ferrocene]-dichloropalladium(II) (10.80 mg, 0.015 mmol) in 1,4-dioxane (10.93 mL) and saturated aqueous sodium bicarbonate (5.47 mL) was heated at 140 °C for 30 min. The reaction was filtered through celite. TFAA (1 mL) was added, and the solution was concentrated in vacuo. The residue was dissolved in DMF (2 mL), filtered, and purified by prep HPLC (acidic mode) to give (S)-6a-methyl-2-(7-methyl-1H-indol-3-yl)-5-(tetrahydro-2H-pyran-4-yl)-6a,7,9,10-tetrahydro-[1,4]oxazino[3,4-h]pteridin-6(5H)-one TFA salt (74 mg, 46%) as an off-white solid. LRMS m/z: $[M+H]^+$ calcd for $C_{24}H_{27}N_5O_3$, 434.2; found, 434.4. ^1H NMR (400 MHz, CHLOROFORM-d) δ ppm 1.63 (s, 3 H) 1.66 - 1.75 (m, 2 H) 2.58 (s, 3 H) 2.63 - 2.82 (m, 2 H) 3.47 - 3.66 (m, 3 H) 3.69 - 3.83 (m, 2 H) 4.09 - 4.19 (m, 2 H) 4.22

- 4.39 (m, 3 H) 4.57 (d, J=13.6 Hz, 1 H) 7.16 (d, J=7.3 Hz, 1 H) 7.25 - 7.26 (m, 1 H) 8.14 (d, J=8.1 Hz, 1 H) 8.32 (br. s., 1 H) 9.07 (br. s., 1 H) 9.19 (br. s., 1 H).

Cmpd 13a

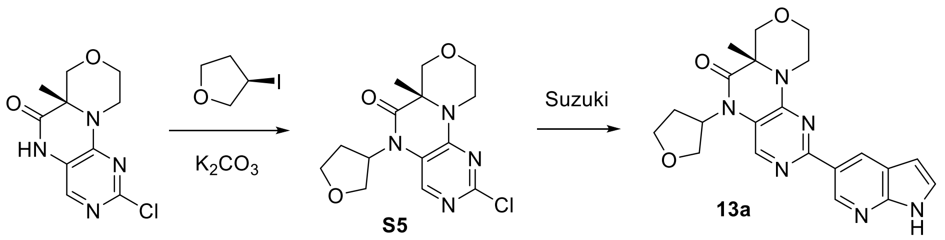

S5. To a suspension of (S)-2-chloro-6a-methyl-6a,7,9,10-tetrahydro-[1,4]oxazino[3,4-h]pteridin-6(5H)-one (125 mg, 0.49 mmol) and potassium carbonate (170 mg, 1.23 mmol) in ACN (5.0 mL) was added (R)-3-iodotetrahydrofuran (0.135 mL, 1.23 mmol) in one portion at 23 °C. The reaction mixture was stirred at 90 °C for 16 h. The solution was diluted with ethyl acetate (20.0 mL) and partitioned with 0.5 M monobasic potassium phosphate (10 mL, pH 4.6). The layers were separated, and the organic phase was washed with brine (10 mL), dried with sodium sulfate, and concentrated in vacuo. The organic extract was dissolved in ethanol (1.5 mL) and treated with 3 M aqueous hydrochloric acid (0.5 mL) for 30 min at 60 °C to cleave the O-alkylated by-product. The crude was purified by silica gel chromatography eluted with DCM/methanol (0-10% methanol) to give (S)-2-chloro-6a-methyl-5-((±)-tetrahydrofuran-3-yl)-6a,7,9,10-tetrahydro-[1,4]oxazino[3,4-h]pteridin-6(5H)-one (65 mg, 41%) as a tan solid, 2:1 mixture of diastereomers by NMR. LRMS m/z: $[M+H]^+$ calcd for $C_{14}H_{17}ClN_4O_3$, 325.1; found, 325.1. ^1H NMR (400 MHz, DMSO-d6) δ ppm 1.31 - 1.37 (m, 3 H) 1.93 - 2.22 (m, 2 H) 3.14 - 3.24 (m, 1 H) 3.48 - 3.57 (m, 1 H) 3.59 - 3.71 (m, 2 H) 3.75 - 4.03 (m, 5 H) 4.13 - 4.22 (m, 1 H) 5.24 - 5.62 (m, 1 H) 8.21 - 8.25 (m, 1 H).

13a. A mixture of (S)-2-chloro-6a-methyl-5-((S)-tetrahydrofuran-3-yl)-6a,7,9,10-tetrahydro-[1,4]oxazino[3,4-h]pteridin-6(5H)-one (32.4 mg, 0.100 mmol), 5-(4,4,5,5-Tetramethyl-1,3,2-dioxaborolan-2-yl)-1H-pyrrolo[2,3-b]pyridine (36.5 mg, 0.150 mmol), $PdCl_2$(dppf)-DCM adduct(4.07 mg, 4.99 µmol), and tribasic potassium phosphate(52.9 mg, 0.249 mmol) in 1,4-dioxane (360 µL) and water (180 µL) was heated in a microwave at 110 °C for 30 m. The crude reaction was concentrated in vacuo. The crude was reconstituted in DMSO (2.0 mL), filtered, and purified by prep HPLC (acidic mode) to give the TFA salt. The TFA salt was dissolved in methanol (3 mL) and passed through a 500 mg StratoSpheres SPE cartridge (PL-HCO3 MP SPE) to remove TFAA. The cartridge was rinsed with methanol (3 x 2 mL) and the filtrate was dried in vacuo to give (S)-6a-methyl-2-(1H-pyrrolo[2,3-b]pyridin-5-yl)-5-((S)-tetrahydrofuran-3-yl)-6a,7,9,10-tetrahydro-[1,4]oxazino[3,4-h]pteridin-6(5H)-one (11 mg, 27% yield) as an off-white solid. LRMS m/z: $[M+H]^+$ calcd for $C_{21}H_{22}N_6O_3$, 407.2; found, 407.2. ^1H NMR (400 MHz, DMSO-d6) δ ppm 1.29 - 1.36 (m, 3 H) 2.01 - 2.30 (m, 2 H) 3.24 - 3.31 (m, 1 H) 3.56 - 3.76 (m, 3 H) 3.82 - 3.90 (m, 1 H) 3.93 - 4.10 (m, 3 H) 4.15 - 4.28 (m, 2 H) 5.27 - 5.69 (m, 1 H) 6.53 - 6.58 (m, 1 H) 7.50 - 7.54 (m, 1 H) 8.45 - 8.50 (m, 1 H) 8.81 - 8.85 (m, 1 H) 9.18 - 9.22 (m, 1 H) 11.83 (br. s., 1 H).

Cmpd 13b

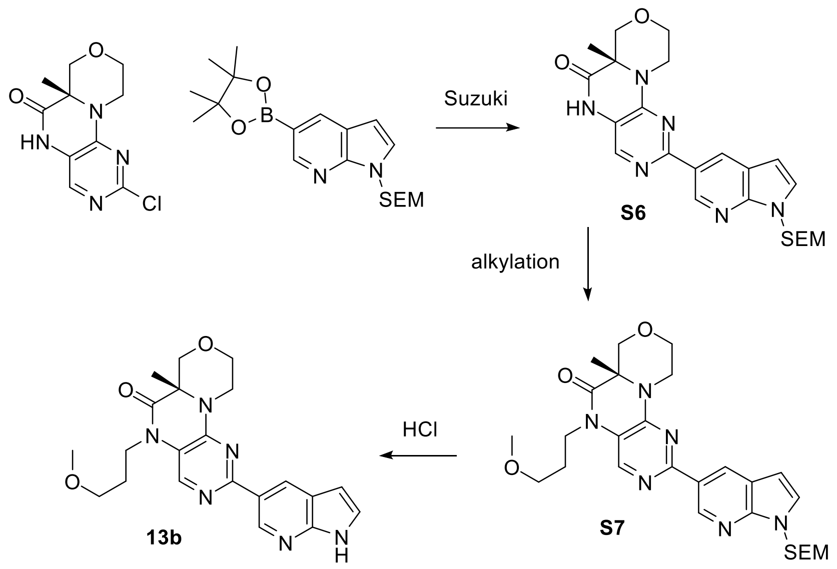

S6. A mixture of (S)-2-chloro-6a-methyl-6a,7,9,10-tetrahydro-[1,4]oxazino[3,4-h]pteridin-6(5H)-one[Hicks-2013] (250 mg, 0.982 mmol), 5-(4,4,5,5-tetramethyl-1,3,2-dioxaborolan-2-yl)-1-((2-(trimethylsilyl)ethoxy)methyl)-1H-pyrrolo[2,3-b]pyridine (735 mg, 1.963 mmol), $PdCl_2$(dppf)-DCM adduct(40.1 mg, 0.049 mmol), and tribasic potassium phosphate (521 mg, 2.454 mmol) in 1,4-dioxane (3.0 mL) and water (1.5 mL) was heated in a microwave at 110 °C for 1 h. The crude reaction was diluted with ethyl acetate (10 mL) and partitioned with 1 M aqueous monobasic potassium phosphate (5 mL, pH 4.6). The layers were separated, and the organic phase was washed with brine (5 mL), dried with sodium sulfate, and concentrated in vacuo. The crude purified by silica gel chromatography eluted with DCM/methanol (0-5% methanol) to give (S)-6a-methyl-2-(1-((2-(trimethylsilyl)ethoxy)methyl)-1H-pyrrolo[2,3-b]pyridin-5-yl)-6a,7,9,10-tetrahydro-[1,4]oxazino[3,4-h]pteridin-6(5H)-one (256 mg, 56%) as a yellow-orange solid. LRMS m/z: $[M+H]^+$ calcd for $C_{23}H_{30}N_6O_3Si$, 467.2; found, 467.3. 1H NMR (400 MHz, DMSO-d6) d ppm -0.11 (s, 9 H) 0.78 - 0.87 (m, 2 H) 1.42 (s, 3 H) 3.17 - 3.33 (m, 1 H) 3.48 - 3.55 (m, 1 H) 3.59 (td, J=12.06, 2.91 Hz, 1 H) 3.65 - 3.73 (m, 1 H) 3.86 - 4.00 (m, 3 H) 4.06 (dd, J=11.49, 3.66 Hz, 1 H) 4.29 (dd, J=13.64, 2.27 Hz, 1 H) 5.64 (s, 1 H) 6.64 (d, J=3.54 Hz, 1 H) 7.68 (d, J=3.54 Hz, 1 H) 7.95 (s, 1 H) 8.82 (d, J=2.02 Hz, 1 H) 9.21 (d, J=2.02 Hz, 1 H) 10.91 (s, 1 H).

S7. A solution of (S)-6a-methyl-2-(1-((2-(trimethylsilyl)ethoxy)methyl)-1H-pyrrolo[2,3-b]pyridin-5-yl)-6a,7,9,10-tetrahydro-[1,4]oxazino[3,4-h]pteridin-6(5H)-one (50.0 mg, 0.107 mmol) in DMF (1.0 mL) was cooled to 0 °C. Sodium tert-butoxide (25.7 mg, 0.268 mmol) was added followed by 1-bromo-3-methoxypropane (0.030 mL, 0.268 mmol) in one portion. The reaction mixture was stirred at 0 °C for one hour, warmed slowly to room temperature, and stirred at 40 °C for 3 h. The reaction mixture was cooled to 20 °C and stirred for 3 days. The crude reaction was diluted with ethyl acetate (2.0 mL) and partitioned with 0.5 M aqueous monobasic potassium phosphate (2 mL, pH 4.6). The layers were separated, and the aqueous phase was washed with ethyl acetate (2 x 1 mL). The organic extracts were combined, washed with brine (1 mL), dried with sodium sulfate, and concentrated in vacuo. The crude material was purified by silica gel chromatography eluted with DCM/methanol (0-5% methanol) to give (S)-5-(3-methoxypropyl)-6a-methyl-2-(1-((2-(trimethylsilyl)ethoxy)methyl)-1H-pyrrolo[2,3-b]pyridin-5-

yl)-6a,7,9,10-tetrahydro-[1,4]oxazino[3,4-h]pteridin-6(5H)-one (28 mg, 49%) as a yellow oil. LRMS m/z: $[M+H]^+$ calcd for $C_{27}H_{38}N_6O_4Si$, 539.3; found, 539.3.

13b. To a solution of (S)-5-(3-methoxypropyl)-6a-methyl-2-(1-((2-(trimethylsilyl)ethoxy)methyl)-1H-pyrrolo[2,3-b]pyridin-5-yl)-6a,7,9,10-tetrahydro-[1,4]oxazino[3,4-h]pteridin-6(5H)-one (28.4 mg, 0.053 mmol) in ethanol (1.0 mL) was added aqueous 3 M hydrochloric acid (0.088 mL, 0.264 mmol) at 20 °C. The reaction was heated at 90 °C for 16 h. The reaction mixture was cooled to 23 °C and an additional portion of ethanol (1.0 mL) was added followed by aqueous 3 M hydrochloric acid (0.088 mL, 0.264 mmol). The reaction was stirred at 90 °C for an additional 5 h. The reaction mixture was cooled to 23 °C and an additional portion of ethanol (1.0 mL) was added followed by aqueous 3 M hydrochloric acid (0.176 mL, 0.527 mmol). The reaction was stirred at 90 °C for an additional 4 h. The crude mixture was concentrated in vacuo, dissolved in DMSO (1 mL), filtered, rinsed with DMSO, and purified by prep HPLC (acidic mode) to give the product as the TFA salt. The TFA salt was dissolved in methanol (2 mL) and passed through a 100 mg StratoSpheres SPE cartridge (PL-HCO3 MP SPE) to remove TFAA. The cartridge was rinsed with methanol (3 x 1 mL) and the filtrate was dried in vacuo to give (S)-5-(3-methoxypropyl)-6a-methyl-2-(1H-pyrrolo[2,3-b]pyridin-5-yl)-6a,7,9,10-tetrahydro-[1,4]oxazino[3,4-h]pteridin-6(5H)-one (11 mg, 51%) as a white solid. LRMS m/z: $[M+H]^+$ calcd for $C_{21}H_{24}N_6O_3$, 409.2; found, 409.3. ^1H NMR (400 MHz, DMSO-d6) δ ppm 1.37 (s, 3 H) 1.82 (quin, J=6.51 Hz, 2 H) 3.24 (s, 3 H) 3.26 - 3.33 (m, 1 H) 3.39 (t, J=6.06 Hz, 2 H) 3.60 (td, J=12.00, 3.03 Hz, 1 H) 3.69 (d, J=11.37 Hz, 1 H) 3.88 - 4.05 (m, 3 H) 4.08 (dd, J=11.37, 3.54 Hz, 1 H) 4.26 (dd, J=13.77, 2.40 Hz, 1 H) 6.56 (d, J=3.28 Hz, 1 H) 7.52 (d, J=3.54 Hz, 1 H) 8.30 (s, 1 H) 8.82 (d, J=2.02 Hz, 1 H) 9.20 (d, J=2.02 Hz, 1 H) 11.82 (br. s., 1 H).

Cmpd 13c

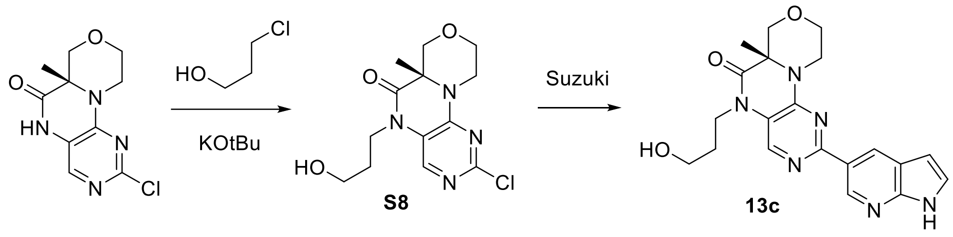

S8. (S)-2-chloro-6a-methyl-6a,7,9,10-tetrahydro-[1,4]oxazino[3,4-h]pteridin-6(5H)-one (150 mg, 0.589 mmol) was dissolved in DMF (5.9 mL) and cooled in an ice bath. Potassium tert-butoxide (72.7 mg, 0.648 mmol) was added and the mixture stirred for 15 min. 3-chloropropan-1-ol (0.055 mL, 0.648 mmol) was added and the ice bath removed. The mixture was heated to 80 °C for 18 h. Reaction was partially concentrated in vacuo. The residue was diluted with ethyl acetate (30mL) and washed with water (15 mL). The aqueous layer was extracted with ethyl acetate (2 x 30 mL). The combined organics were washed with brine (25 mL), dried with magnesium sulfate, and concentrated in vacuo to give (S)-2-chloro-5-(3-hydroxypropyl)-6a-methyl-6a,7,9,10-tetrahydro-[1,4]oxazino[3,4-h]pteridin-6(5H)-one (184 mg, 100%) which was used without further purification. LRMS m/z: $[M+H]^+$ calcd for $C_{13}H_{17}ClN_4O_3$, 313.1; found, 313.1.

13c. A mixture of (S)-2-chloro-5-(3-hydroxypropyl)-6a-methyl-6a,7,9,10-tetrahydro-[1,4]oxazino[3,4-h]pteridin-6(5H)-one (184mg, 0.588 mmol), 5-(4,4,5,5-tetramethyl-1,3,2-dioxaborolan-2-yl)-1H-

pyrrolo[2,3-b]pyridine (287 mg, 1.177 mmol) and $PdCl_2$(dppf) (21.52 mg, 0.029 mmol) in 1,4-dioxane (1.96 mL) and saturated aqueous sodium bicarbonate (0.98 mL) was degassed with nitrogen and heated in the microwave at 115 °C for 30 min. The organic solvent was removed in vacuo. The residue was taken up in 1:1 methanol:DMF (2mL), filtered, and purified by prep HPLC (basic mode) to give (S)-5-(3-hydroxypropyl)-6a-methyl-2-(1H-pyrrolo[2,3-b]pyridin-5-yl)-6a,7,9,10-tetrahydro-[1,4]oxazino[3,4-h]pteridin-6(5H)-one (17 mg, 7%) as a white solid. LRMS m/z: $[M+H]^+$ calcd for $C_{20}H_{22}N_6O_3$, 395.2; found, 395.4. ^1H NMR (400 MHz, DMSO-d6) δ ppm 1.36 (s, 3 H) 1.74 (quin, J=6.7 Hz, 2 H) 3.26 - 3.33 (m, 1 H) 3.48 (d, J=3.3 Hz, 2 H) 3.60 (td, J=12.0, 2.8 Hz, 1 H) 3.69 (d, J=11.4 Hz, 1 H) 3.88 - 4.13 (m, 4 H) 4.26 (dd, J=13.5, 2.1 Hz, 1 H) 4.63 (br. s., 1 H) 6.56 (dd, J=3.5, 1.8 Hz, 1 H) 7.52 (dd, J=3.3, 2.5 Hz, 1 H) 8.34 (s, 1 H) 8.82 (d, J=1.5 Hz, 1 H) 9.20 (d, J=1.5 Hz, 1 H) 11.82 (br. s., 1 H).

Cmpd 13d

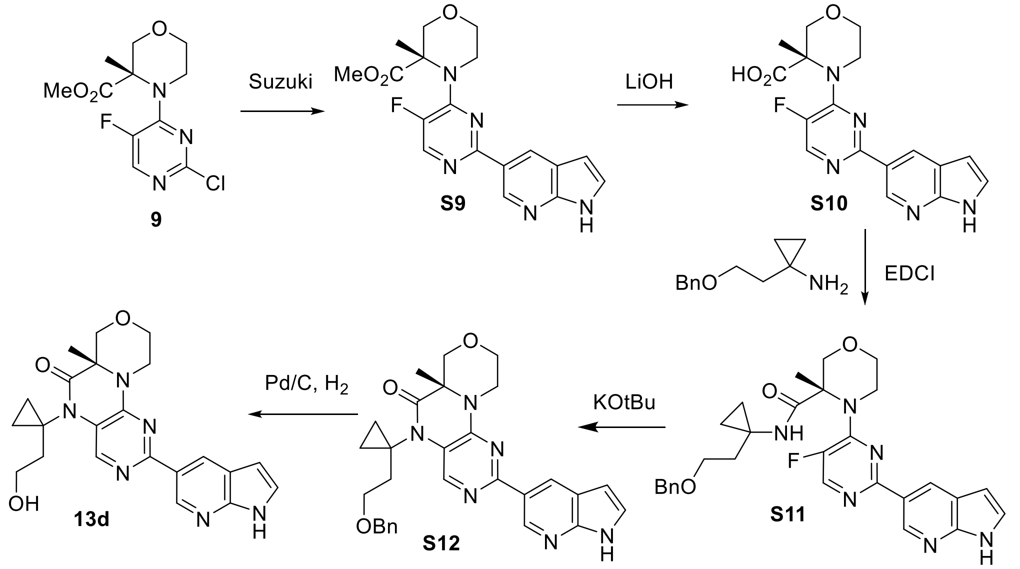

S9. A mixture of (S)-methyl 4-(2-chloro-5-fluoropyrimidin-4-yl)-3-methylmorpholine-3-carboxylate (1.0 g, 3.45 mmol), 5-(4,4,5,5-Tetramethyl-1,3,2-dioxaborolan-2-yl)-1H-pyrrolo[2,3-b]pyridine (1.095 g, 4.49 mmol), $PdCl_2$(dppf)-DCM adduct(0.141 g, 0.173 mmol), and tribasic potassium phosphate(1.832 g, 8.63 mmol) in 1,4-dioxane (12.5 mL) and water (6.25 mL) was heated for 1 h at 100 °C under nitrogen. The reaction mixture was cooled to room temperature, diluted with ethyl acetate (100 mL), washed with saturated ammonium chloride aqueous (100 mL), saturated sodium bicarbonate aqueous (100 mL) and brine (100 mL), dried over magnesium sulfate, and concentrated in vacuo . The crude was purified by silica gel chromatography eluted with ethyl acetate in hexanes (25-100%) to give (S)-methyl 4-(5-fluoro-2-(1H-pyrrolo[2,3-b]pyridin-5-yl)pyrimidin-4-yl)-3-methylmorpholine-3-carboxylate (1.16 g, 91% yield) as a light yellow solid. m/z: $[M+H]^+$ calcd for $C_{18}H_{18}FN_5O_3$, 372.1; found, 372.2. ^1H NMR (400 MHz, DMSO-d6) δ ppm 1.71 (s, 3 H) 3.48 (s, 3 H) 3.52 - 3.62 (m, 1 H) 3.72 - 3.87 (m, 3 H) 3.92 - 4.05 (m, 2 H) 6.56 (dd, J=3.5, 1.8 Hz, 1 H) 7.51 - 7.57 (m, 1 H) 8.52 (d, J=6.3 Hz, 1 H) 8.68 (d, J=1.52 Hz, 1 H) 9.04 (d, J=2.3 Hz, 1 H) 11.85 (br. s., 1 H).

S10. To a suspension of (S)-methyl 4-(5-fluoro-2-(1H-pyrrolo[2,3-b]pyridin-5-yl)pyrimidin-4-yl)-3-methylmorpholine-3-carboxylate (1.157 g, 3.12 mmol) in 1,4-dioxane (23 mL) was added lithium hydroxide (15.58 mL, 15.58 mmol) at 23 °C. The mixture was heated at 100 °C in an oil bath for 9 h. The mixture was cooled to room temperature and neutralized to pH 2-3 with 1 M hydrochloric acid (15.58 mL) to furnish a light yellow suspension. The suspension was concentrated via rotary evaporation to half volume, filtered, rinsed with water, and dried in vacuo to provide (S)-4-(5-fluoro-2-(1H-pyrrolo[2,3-b]pyridin-5-yl)pyrimidin-4-yl)-3-methylmorpholine-3-carboxylic acid (1.10 g, 99% yield) as an off-white solid. LRMS m/z: $[M+H]^+$ calcd for $C_{17}H_{16}FN_5O_3$, 358.1; found, 358.2. ^1H NMR (400 MHz, DMSO-d6) δ ppm 1.67 (s, 3 H) 3.47 - 3.60 (m, 1 H) 3.69 - 3.75 (m, 1 H) 3.76 - 3.88 (m, 2 H) 3.90 - 4.03 (m, 2 H) 6.53 (dd, J=3.28, 1.77 Hz, 1 H) 7.48 - 7.57 (m, 1 H) 8.50 (d, J=6.32 Hz, 1 H) 8.74 (d, J=1.77 Hz, 1 H) 9.10 (d, J=2.02 Hz, 1 H) 11.83 (br. s., 1 H) 12.74 (s, 1 H).

S11. A solution of (S)-4-(5-fluoro-2-(1H-pyrrolo[2,3-b]pyridin-5-yl)pyrimidin-4-yl)-3-methylmorpholine-3-carboxylic acid (100 mg, 0.280 mmol), HOBT (42.9 mg, 0.280 mmol), EDCI (107 mg, 0.560 mmol), 1-(2-(benzyloxy)ethyl)cyclopropanamine[Bertus-2002] (161 mg, 0.840 mmol) and DIPEA (0.171 mL, 0.979 mmol) in DMF (2 mL) was stirred at 20 °C for 2 days. The solution was diluted with ethyl acetate (50 mL), washed with saturated aqueous ammonium chloride (50 mL) and brine (50 mL), dried with magnesium sulfate and concentrated in vacuo to give (S)-N-(1-(2-(benzyloxy)ethyl)cyclopropyl)-4-(5-fluoro-2-(1H-pyrrolo[2,3-b]pyridin-5-yl)pyrimidin-4-yl)-3-methylmorpholine-3-carboxamide (149 mg theoretical, yield calculated on final **13d**). The crude was used without further purification. LRMS m/z: $[M+H]^+$ calcd for $C_{29}H_{31}FN_6O_3$, 531.2; found, 531.4.

S12. To a solution of (S)-N-(1-(2-(benzyloxy)ethyl)cyclopropyl)-4-(5-fluoro-2-(1H-pyrrolo[2,3-b]pyridin-5-yl)pyrimidin-4-yl)-3-methylmorpholine-3-carboxamide (0.149 g, 0.28 mmol) in NMP (2.0 mL) at 20 °C was added 1 M potassium tert-butoxide in THF (0.700 mL, 0.700 mmol) and the solution stirred at 20 °C for 4 h. More 1 M potassium tert-butoxide in THF (420 µL, 1.5 eq) was added and stirring continued at 20 °C for 18 h. More 1 M potassium tert-butoxide in THF (420 µL, 1.5 eq) was added and stirring continued at 20 °C for 3.5 h. More 1 M potassium tert-butoxide in THF (210 µL, 0.75 eq) was added and stirring at 20 °C continued for 2 h. The solution was diluted with ethyl acetate (50 mL), washed with saturated aqueous ammonium chloride (50 mL) and brine (50 mL), dried with magnesium sulfate and concentrated in vacuo to give (S)-5-(1-(2-(benzyloxy)ethyl)cyclopropyl)-6a-methyl-2-(1H-pyrrolo[2,3-b]pyridin-5-yl)-6a,7,9,10-tetrahydro-[1,4]oxazino[3,4-h]pteridin-6(5H)-one (143 mg theoretical, yield calculated on final **13d**). The crude was used without further purification. LRMS m/z: $[M+H]^+$ calcd for $C_{29}H_{30}N_6O_3$, 511.2; found, 511.4.

13d. A slurry of (S)-5-(1-(2-(benzyloxy)ethyl)cyclopropyl)-6a-methyl-2-(1H-pyrrolo[2,3-b]pyridin-5-yl)-6a,7,9,10-tetrahydro-[1,4]oxazino[3,4-h]pteridin-6(5H)-one (143 mg, 0.28 mmol) and palladium on carbon (10 wt% Degussa) (85 mg, 0.799 mmol) in methanol (3 mL) and 4 M hydrochloric acid in 1,4-dioxane (0.490 mL, 1.960 mmol) at 20 °C was stirred under an atmosphere of hydrogen for 7 h. The slurry was filtered through Celite to remove Pd/C and rinsed with methanol. The filtrate was concentrated in vacuo. The residue was taken up in DMSO (1.5 mL) and purified by prep HPLC (acidic mode) to give (S)-5-(1-(2-hydroxyethyl)cyclopropyl)-6a-methyl-2-(1H-pyrrolo[2,3-b]pyridin-5-yl)-6a,7,9,10-tetrahydro-[1,4]oxazino[3,4-h]pteridin-6(5H)-one, TFA salt (32 mg, 21% over 3 steps) as a tan solid. NMR run at high temperature to eliminate rotamers. LRMS m/z: $[M+H]^+$ calcd for $C_{22}H_{24}N_6O_3$, 421.2; found, 421.4. ^1H NMR (500 MHz, DMSO-d6, 85 °C) δ ppm 0.65 - 1.09 (m, 2 H) 1.10 - 1.30 (m, 2

H) 1.39 (s, 3 H) 2.03 (br. s., 2 H) 3.30 (t, J=13.4 Hz, 1 H) 3.50 (br. s., 1 H) 3.53 - 3.77 (m, 4 H) 3.99 (d, J=11.7 Hz, 1 H) 4.07 (dd, J=11.2, 3.9 Hz, 1 H) 4.18 (d, J=12.7 Hz, 1 H) 6.56 (dd, J=3.4, 2.0 Hz, 1 H) 7.44 - 7.50 (m, 1 H) 8.53 (s, 1 H) 8.81 (d, J=2.0 Hz, 1 H) 9.19 (d, J=1.5 Hz, 1 H) 11.54 (br. s., 1 H)

Cmpd 13e

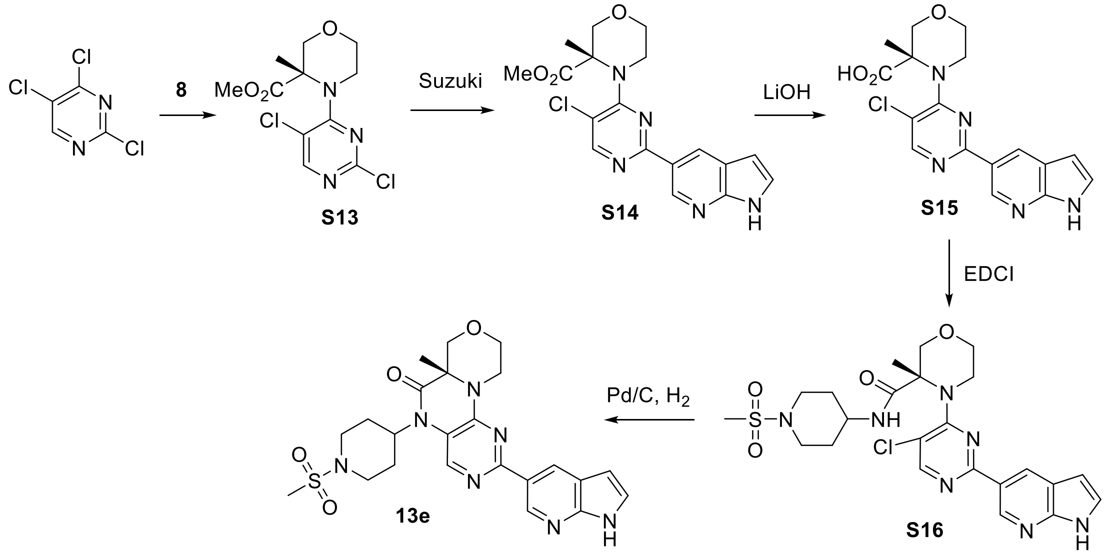

S13. A solution of (S)-3-(methoxycarbonyl)-3-methylmorpholin-4-ium (7,7-dimethyl-2-oxobicyclo[2.2.1]heptan-1-yl)methanesulfonate[Hicks-2013] (10.24 g, 26.2 mmol), 2,4,5-trichloropyrimidine (2.500 mL, 21.81 mmol) and DIPEA (15.23 mL, 87 mmol) in NMP (15 mL) was heated at 70 °C for 2 days. The solution was diluted with ethyl acetate (200 mL), washed with saturated aqueous ammonium chloride (2x250 mL), saturated aqueous sodium bicarbonate (250 mL) and brine (250 mL), dried with magnesium sulfate and concentrated in vacuo. The crude was purified by silica gel chromatography eluted with 0 to 40% ethyl acetate in hexanes to give (S)-methyl 4-(2,5-dichloropyrimidin-4-yl)-3-methylmorpholine-3-carboxylate (2.96 g, 44%) as an orange oil. LRMS m/z: $[M+H]^+$ calcd for $C_{11}H_{13}Cl_2N_3O_3$, 305.0; found, 306.1. ^{1}H NMR (400 MHz, chloroform-d) δ ppm 1.64 (s, 3 H) 3.63 - 3.72 (m, 2 H) 3.74 (s, 3 H) 3.88 - 3.97 (m, 3 H) 4.13 - 4.22 (m, 1 H) 8.23 (s, 1 H).

S14. A mixture of (S)-methyl 4-(2,5-dichloropyrimidin-4-yl)-3-methylmorpholine-3-carboxylate (1000 mg, 3.27 mmol), 5-(4,4,5,5-tetramethyl-1,3,2-dioxaborolan-2-yl)-1H-pyrrolo[2,3-b]pyridine (797 mg, 3.27 mmol) , $PdCl_2$(dppf)-DCM adduct(133 mg, 0.163 mmol) and tribasic potassium phosphate(2080 mg, 9.80 mmol) in 1,4-dioxane (6 mL) and water (3.00 mL) was heated in the microwave at 90 °C for 1 h. The solution was diluted with ethyl acetate (100 mL), washed with saturated aqueous ammonium chloride (2 x 100 mL) and brine (100 mL), dried with magnesium sulfate, and concentrated in vacuo. The crude was purified by silica gel chromatography eluted with 10 to 80% ethyl acetate in hexanes to give (S)-methyl 4-(5-chloro-2-(1H-pyrrolo[2,3-b]pyridin-5-yl)pyrimidin-4-yl)-3-methylmorpholine-3-carboxylate (510 mg, 40%) as a light yellow solid. LRMS m/z: $[M+H]^+$ calcd for $C_{18}H_{18}ClN_5O_3$, 388.1; found, 388.2. ^{1}H NMR (400 MHz, DMSO-d6) δ ppm 1.68 (s, 3 H) 3.53 (s, 3 H) 3.62 (ddd, J=13.3, 7.6, 3.4 Hz, 1 H) 3.70 (d, J=11.4 Hz, 1 H) 3.79 - 3.95 (m, 3 H) 4.09 (ddd, J=13.3, 5.3, 3.2 Hz, 1 H) 6.58 (dd, J=3.5, 1.8 Hz, 1 H) 7.56 (dd, J=3.3, 2.5 Hz, 1 H) 8.63 (s, 1 H) 8.69 - 8.74 (m, 1 H) 9.07 (d, J=2.0 Hz, 1 H) 11.91 (br. s., 1 H).

S15. A solution of (S)-methyl 4-(5-chloro-2-(1H-pyrrolo[2,3-b]pyridin-5-yl)pyrimidin-4-yl)-3-methylmorpholine-3-carboxylate (505 mg, 1.302 mmol) in 1,4-dioxane (7.0 mL) and 1.0 M aqueous lithium hydroxide solution (7.03 mL, 7.03 mmol) was heated at 100 °C for 10 h. The solution was poured into water (25 mL) and acidified with 1 M aqueous hydrochloric acid (5.4 mL) to pH 4 which formed white precipitate. The aqueous layer was extracted with methylene chloride (50 mL). The organic layer was washed with brine (50 mL), dried with magnesium sulfate, and concentrated in vacuo to give (S)-4-(5-chloro-2-(1H-pyrrolo[2,3-b]pyridin-5-yl)pyrimidin-4-yl)-3-methylmorpholine-3-carboxylic acid (372 mg, 0.995 mmol, 76 % yield) as a white solid. LRMS m/z: $[M+H]^+$ calcd for $C_{17}H_{16}ClN_5O_3$, 374.1; found, 374.2. ^1H NMR (400 MHz, DMSO-d6) δ ppm 1.67 (s, 3 H) 3.50 - 3.56 (m, 1 H) 3.67 (d, J=11.1 Hz, 1 H) 3.77 - 3.94 (m, 3 H) 4.03 - 4.13 (m, 1 H) 6.55 (dd, J=3.5, 1.8 Hz, 1 H) 7.51 - 7.58 (m, 1 H) 8.61 (s, 1 H) 8.77 (d, J=1.5 Hz, 1 H) 9.13 (d, J=2.0 Hz, 1 H) 11.89 (br. s., 1 H) 12.78 (s, 1 H)

S16. A solution of (S)-4-(5-chloro-2-(1H-pyrrolo[2,3-b]pyridin-5-yl)pyrimidin-4-yl)-3-methylmorpholine-3-carboxylic acid (40 mg, 0.107 mmol), EDCI (30.8 mg, 0.161 mmol), HOBT (24.58 mg, 0.161 mmol), DIPEA (0.065 mL, 0.375 mmol) and 1-(methylsulfonyl)piperidin-4-amine hydrochloride (46.0 mg, 0.214 mmol) in DMF (0.7 mL) was stirred at 20 °C for 3 h. The solution was diluted with ethyl acetate (50 mL), washed with saturated aqueous ammonium chloride (50 mL) and brine (50 mL), dried with magnesium sulfate and concentrated in vacuo to give (S)-4-(5-chloro-2-(1H-pyrrolo[2,3-b]pyridin-5-yl)pyrimidin-4-yl)-3-methyl-N-(1-(methylsulfonyl)piperidin-4-yl)morpholine-3-carboxamide (57 mg theoretical, yield determined in next step) which was used without further purification. LRMS m/z: $[M+H]^+$ calcd for $C_{23}H_{28}ClN_7O_4S$, 534.2; found, 534.3.

13e. To a solution of (S)-4-(5-chloro-2-(1H-pyrrolo[2,3-b]pyridin-5-yl)pyrimidin-4-yl)-3-methyl-N-(1-(methylsulfonyl)piperidin-4-yl)morpholine-3-carboxamide (57 mg, 0.107 mmol) in DMF (0.8 mL) was added 1 M NaHMDS in THF (0.320 mL, 0.320 mmol) and the solution heated at 90 °C for 14 h. The solution was diluted with 0.3 mL DMSO and purified on prep HPLC (basic mode) to give (S)-6a-methyl-5-(1-(methylsulfonyl)piperidin-4-yl)-2-(1H-pyrrolo[2,3-b]pyridin-5-yl)-6a,7,9,10-tetrahydro-[1,4]oxazino[3,4-h]pteridin-6(5H)-one (4 mg, 7.5% over 2 steps) as a white solid. LRMS m/z: $[M+H]^+$ calcd for $C_{23}H_{27}N_7O_4S$, 498.2; found, 498.3. ^1H NMR (400 MHz, DMSO-d6) δ ppm 1.30 (s, 3 H) 1.72 - 1.82 (m, 1 H) 1.88 (d, J=12.9 Hz, 1 H) 2.58 - 2.72 (m, 1 H) 2.85 (d, J=9.9 Hz, 1 H) 2.92 (s, 3 H) 2.97 - 3.08 (m, 1 H) 3.27 (td, J=13.0, 4.3 Hz, 1 H) 3.57 - 3.74 (m, 4 H) 3.97 (d, J=11.4 Hz, 2 H) 4.08 (dd, J=11.9, 3.5 Hz, 1 H) 4.18 (d, J=11.1 Hz, 1 H) 4.31 - 4.41 (m, 1 H) 6.57 (dd, J=3.4, 1.9 Hz, 1 H) 7.49 - 7.57 (m, 1 H) 8.56 (s, 1 H) 8.83 (d, J=2.0 Hz, 1 H) 9.20 (d, J=2.0 Hz, 1 H) 11.85 (br. s., 1 H)

Cmpd 14a

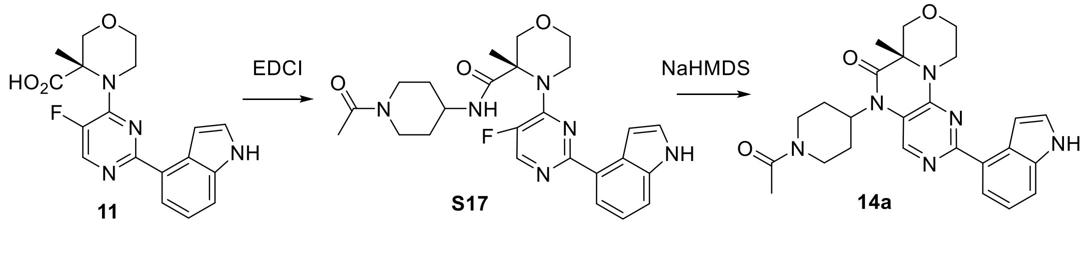

S17. A solution of (S)-4-(5-fluoro-2-(1H-indol-4-yl)pyrimidin-4-yl)-3-methylmorpholine-3-carboxylic acid (50 mg, 0.140 mmol), HOBT (32.2 mg, 0.210 mmol), EDCI (40.3 mg, 0.210 mmol), 1-(4-aminopiperidin-1-yl)ethanone (80 mg, 0.561 mmol) and DIPEA (0.061 mL, 0.351 mmol) in DMF (1 mL) was stirred at 20 °C for 22 h. The solution was diluted with ethyl acetate (50 mL), washed with saturated aqueous ammonium chloride (50 mL) and brine (50 mL), dried with magnesium sulfate and concentrated in vacuo to give (S)-N-(1-acetylpiperidin-4-yl)-4-(5-fluoro-2-(1H-indol-4-yl)pyrimidin-4-yl)-3-methylmorpholine-3-carboxamide (67 mg theoretical, yield calculated in next step) which was used without further purification. LRMS m/z: $[M+H]^+$ calcd for $C_{25}H_{29}FN_6O_3$, 481.2; found, 481.4.

14a. To a solution of (S)-N-(1-acetylpiperidin-4-yl)-4-(5-fluoro-2-(1H-indol-4-yl)pyrimidin-4-yl)-3-methylmorpholine-3-carboxamide (67 mg, 0.139 mmol) in DMF (0.8 mL) was added 1 M NaHMDS in THF (0.446 mL, 0.446 mmol) and the solution stirred at 20 °C for 1 h. The reaction was quenched with glacial acetic acid (40 µL) and water (40 µL) and volatile solvents were removed in vacuo. The solution was diluted with 1 mL DMSO and purified on prep HPLC (basic mode) to give (S)-5-(1-acetylpiperidin-4-yl)-2-(1H-indol-4-yl)-6a-methyl-6a,7,9,10-tetrahydro-[1,4]oxazino[3,4-h]pteridin-6(5H)-one (21 mg, 33% over 2 steps) as a yellow solid. LRMS m/z: $[M+H]^+$ calcd for $C_{25}H_{28}N_6O_3$, 461.2; found, 461.4. ^1H NMR (400 MHz, DMSO-d6) δ ppm 1.32 (s, 3 H) 1.63 - 1.88 (m, 2 H) 2.05 (s, 3 H) 2.14 - 2.48 (m, 2 H) 2.52 - 2.80 (m, 1 H) 3.05 - 3.40 (m, 2 H) 3.57 - 3.72 (m, 2 H) 3.85 - 4.00 (m, 2 H) 4.06 - 4.20 (m, 2 H) 4.50 (d, J=7.3 Hz, 2 H) 7.16 - 7.24 (m, 1 H) 7.35 (br. s., 1 H) 7.47 (t, J=2.8 Hz, 1 H) 7.55 (d, J=8.1 Hz, 1 H) 8.05 (d, J=7.1 Hz, 1 H) 8.64 (d, J=2.5 Hz, 1 H) 11.31 (br. s., 1 H).

Cmpd 14b

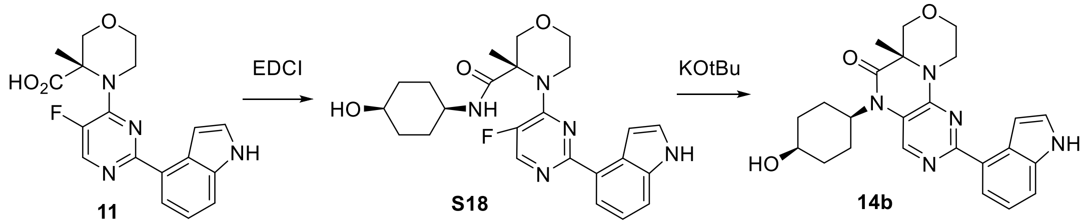

S18. A solution of (S)-4-(5-fluoro-2-(1H-indol-4-yl)pyrimidin-4-yl)-3-methylmorpholine-3-carboxylic acid (3100 mg, 8.70 mmol), HOBT (1332 mg, 8.70 mmol), EDCI (2502 mg, 13.05 mmol), (1s,4s)-4-aminocyclohexanol hydrochloride (1715 mg, 11.31 mmol) and DIPEA (3.80 mL, 21.75 mmol) in DMF (15 mL) was stirred at 20 °C for 4 days. The solution was diluted with ethyl acetate (250 mL), washed with saturated aqueous ammonium chloride (200 mL) and brine (200 mL), dried with magnesium sulfate and concentrated in vacuo to give (S)-4-(5-fluoro-2-(1H-indol-4-yl)pyrimidin-4-yl)-N-((1s,4R)-4-hydroxycyclohexyl)-3-methylmorpholine-3-carboxamide (3.95 g theoretical, yield determined in next step) which was used without further purification. LRMS m/z: $[M+H]^+$ calcd for $C_{24}H_{28}FN_5O_3$, 454.2; found, 454.4.

14b. To a solution of (S)-4-(5-fluoro-2-(1H-indol-4-yl)pyrimidin-4-yl)-N-((1s,4R)-4-hydroxycyclohexyl)-3-methylmorpholine-3-carboxamide (3.95 g, 8.70 mmol) in NMP (30 mL) at 0 °C was added 1.0 M potassium tert-butoxide in THF (30.5 mL, 30.5 mmol) and the solution stirred at 0 °C for 1.5 h. The reaction was concentrated in vacuo to remove THF and then quenched with glacial acetic acid (1.245

mL, 21.75 mmol) and water (5 mL). The viscous solution was poured into water (400 mL) and extracted with ethyl acetate (2 x 400 mL). The combined organics were washed with brine (600 mL), dried with magnesium sulfate, and concentrated in vacuo. The crude was purified by silica gel chromatography eluted with 10 to 100% ethyl acetate in hexanes to give (S)-5-((1s,4R)-4-hydroxycyclohexyl)-2-(1H-indol-4-yl)-6a-methyl-6a,7,9,10-tetrahydro-[1,4]oxazino[3,4-h]pteridin-6(5H)-one (1.08 g, 29% over 2 steps) as a white solid. LRMS m/z: $[M+H]^+$ calcd for $C_{24}H_{27}N_5O_3$, 434.2; found, 434.4. ^1H NMR (400 MHz, DMSO-d6) δ ppm 1.28 (s, 3 H) 1.35 (d, J=11.6 Hz, 1 H) 1.42 - 1.69 (m, 3 H) 1.79 (d, J=12.1 Hz, 2 H) 2.53 - 2.73 (m, 2 H) 3.24 - 3.32 (m, 1 H) 3.57 - 3.73 (m, 2 H) 3.89 (br. s., 1 H) 3.98 (d, J=11.6 Hz, 1 H) 4.06 - 4.17 (m, 2 H) 4.45 - 4.61 (m, 2 H) 7.18 (t, J=7.8 Hz, 1 H) 7.39 (t, J=2.1 Hz, 1 H) 7.44 (t, J=2.8 Hz, 1 H) 7.51 (d, J=8.1 Hz, 1 H) 8.09 (dd, J=7.6, 1.0 Hz, 1 H) 8.58 (s, 1 H) 11.25 (br. s., 1 H).

Cmpd 14c

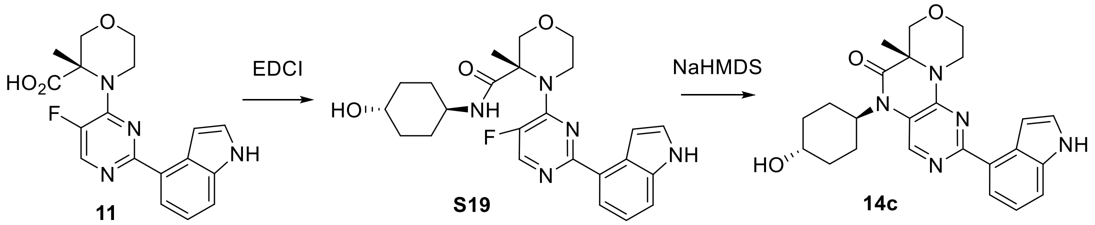

S19. A solution of (S)-4-(5-fluoro-2-(1H-indol-4-yl)pyrimidin-4-yl)-3-methylmorpholine-3-carboxylic acid (40 mg, 0.112 mmol), EDC (32.3 mg, 0.168 mmol), HOBT (25.8 mg, 0.168 mmol), DIPEA (0.069 mL, 0.393 mmol) and (1r,4r)-4-aminocyclohexanol hydrochloride (34.0 mg, 0.224 mmol) in DMF (1.0 mL) was stirred at 20 °C for 19 h. The solution was diluted with ethyl acetate (50 mL), washed with saturated aqueous ammonium chloride (50 mL) and brine (50 mL), dried with magnesium sulfate and concentrated in vacuo to give (S)-4-(5-fluoro-2-(1H-indol-4-yl)pyrimidin-4-yl)-N-((1r,4S)-4-hydroxycyclohexyl)-3-methylmorpholine-3-carboxamide (51 mg theoretical, yield determined in next step) which was used without further purification. LRMS m/z: $[M+H]^+$ calcd for $C_{24}H_{28}FN_5O_3$, 454.2; found, 454.4.

14c. To a solution of (S)-4-(5-fluoro-2-(1H-indol-4-yl)pyrimidin-4-yl)-N-((1r,4S)-4-hydroxycyclohexyl)-3-methylmorpholine-3-carboxamide (48.1 mg, 0.106 mmol) in DMF (0.8 mL) was added 1 M NaHMDS in THF (0.318 mL, 0.318 mmol) and the solution stirred at 20 °C for 1.5 h. The reaction was quenched with glacial acetic acid (0.024 mL, 0.424 mmol) and the volatile organics were removed in vacuo. The solution was diluted with 0.3 mL DMSO and purified by prep HPLC (basic mode) to give (S)-5-((1r,4S)-4-hydroxycyclohexyl)-2-(1H-indol-4-yl)-6a-methyl-6a,7,9,10-tetrahydro-[1,4]oxazino[3,4-h]pteridin-6(5H)-one (24 mg, 52% over 2 steps) as a yellow solid. LRMS m/z: $[M+H]^+$ calcd for $C_{24}H_{27}N_5O_3$, 434.2; found, 434.4. ^1H NMR (400 MHz, DMSO-d6) δ ppm 1.26 (s, 3 H) 1.27 - 1.38 (m, 1 H) 1.39 - 1.52 (m, 1 H) 1.68 (br d, J=11.29 Hz, 2 H) 1.91 (br s, 2 H) 2.24 - 2.39 (m, 1 H) 2.39 - 2.49 (m, 1 H) 3.29 (td, J=12.96, 3.95 Hz, 1 H) 3.46 - 3.57 (m, 1 H) 3.58 - 3.72 (m, 2 H) 3.97 (d, J=11.54 Hz, 1 H) 4.02 - 4.15 (m, 2 H) 4.21 - 4.34 (m, 1 H) 4.62 (d, J=4.14 Hz, 1 H) 7.18 (t, J=7.78 Hz, 1 H) 7.36 - 7.47 (m, 2 H) 7.52 (d, J=8.03 Hz, 1 H) 8.10 (d, J=7.28 Hz, 1 H) 8.58 (s, 1 H) 11.24 (br s, 1 H); ^{13}C NMR (101 MHz, DMSO-d6) δ ppm 15.6, 25.5, 27.0, 34.4, 34.8, one peak under DMSO multiplet, 54.9, 56.7, 65.5, 68.0, 71.0, 103.3, 113.5, 118.6, 120.1, 120.4, 126.0, 126.1, 128.7, 137.0, 139.6, 151.7, 159.3, 166.7; ^{13}C NMR (101 MHz, DMF-d7) δ ppm 15.6, 26.1, 27.7, two peaks under DMF multiplet, 39.6, 55.9, 57.5, 66.3, 69.0, 71.9, 104.2, 113.8, 119.5, 120.7, 120.8,

126.3, 126.9, 129.5, 138.0, 139.9, 152.5, 160.2, 167.6; mp: 306-307 °C. Elemental Analysis: Anal. Calcd for $C_{24}H_{27}N_5O_3$: C, 66.50; H, 6.28; N, 16.15. Found: C, 66.47; H, 6.41; N, 15.88. $[\alpha]_D^{24}$ +62 (c 0.40, 1,4-dioxane).

Cmpd 15a

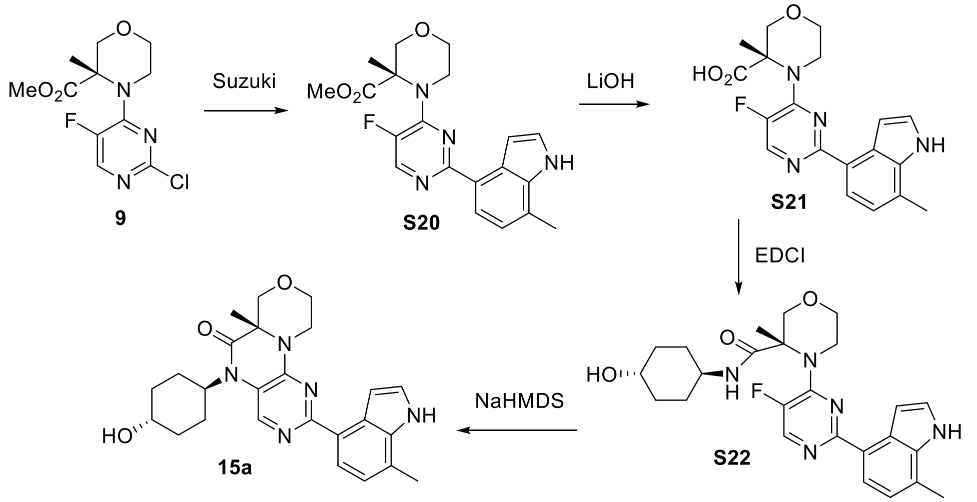

S20. A mixture of 7-methyl-4-(4,4,5,5-tetramethyl-1,3,2-dioxaborolan-2-yl)-1H-indole (888 mg, 3.45 mmol), $PdCl_2$(dppf) (63 mg, 0.086 mmol) and (S)-methyl 4-(2-chloro-5-fluoropyrimidin-4-yl)-3-methylmorpholine-3-carboxylate (500 mg, 1.73 mmol) in 1,4-dioxane (5.75 mL) and saturated aqueous sodium bicarbonate (2.87 mL) was heated in the microwave at 115 °C for 1 h. The volatile solvent was removed in vacuo. The residue was partitioned between water (25 mL) and ethyl acetate (25 mL). The organic layer was separated, and the aqueous layer was saturated with sodium chloride. The aqueous layer was extracted with ethyl acetate (2 x 25 mL). The combined organics were washed with brine (25 mL), dried with magnesium sulfate, and concentrated in vacuo. The crude was purified by column chromatography (20-30% ethyl acetate/hexanes) to give (S)-methyl 4-(5-fluoro-2-(7-methyl-1H-indol-4-yl)pyrimidin-4-yl)-3-methylmorpholine-3-carboxylate (522 mg, 79%) as a white solid. LRMS m/z: $[M+H]^+$ calcd for $C_{20}H_{21}FN_4O_3$, 385.2; found, 385.3. ^{1}H NMR (400 MHz, DMSO-d6) δ ppm 1.69 (s, 3 H) 2.53 (s, 3 H) 3.50 (s, 3 H) 3.53 - 3.63 (m, 1 H) 3.69 - 3.88 (m, 3 H) 3.89 - 4.01 (m, 2 H) 6.97 - 7.03 (m, 1 H) 7.37 - 7.45 (m, 2 H) 7.79 (d, J=7.6 Hz, 1 H) 8.57 (d, J=5.8 Hz, 1 H) 11.23 (br. s., 1 H).

S21. A mixture of (S)-methyl 4-(5-fluoro-2-(7-methyl-1H-indol-4-yl)pyrimidin-4-yl)-3-methylmorpholine-3-carboxylate (508 mg, 1.32 mmol) in 1 M aqueous lithium hydroxide (11.9 mL, 11.9 mmol) and 1,4-dioxane (10 mL) was heated at reflux for 6 h. The volatile solvents were removed in vacuo and the remaining aqueous layer was extracted with ether (30 mL). The aqueous layer was acidified to pH ca. 2-3 where a precipitate formed. The aqueous layer was extracted with ethyl acetate (3 x 50 mL). The combined organics were washed with brine (25 mL), dried with magnesium sulfate, and concentrated in vacuo to give (S)-4-(5-fluoro-2-(7-methyl-1H-indol-4-yl)pyrimidin-4-yl)-3-methylmorpholine-3-carboxylic acid (489 mg, 100%) as a yellow-brown oil. LRMS m/z: $[M+H]^+$ calcd for $C_{19}H_{19}FN_4O_3$, 371.1; found, 371.3. ^{1}H NMR (400 MHz, DMSO-d6) δ ppm 1.65 (s, 3 H) 2.51 - 2.54 (m, 3 H) 3.54 (s, 1 H) 3.68 (d,

J=11.37 Hz, 1 H) 3.78 - 3.86 (m, 2 H) 3.87 - 3.98 (m, 2 H) 6.91 - 6.99 (m, 1 H) 7.35 - 7.43 (m, 2 H) 7.88 (d, J=7.6 Hz, 1 H) 8.54 (d, J=6.1 Hz, 1 H) 11.20 (br. s., 1 H) 12.64 (br. s., 1 H).

S22. To a solution of HOBT (78 mg, 0.506 mmol), EDCI (97 mg, 0.506 mmol), DIPEA (118 µl, 0.675 mmol) and (S)-4-(5-fluoro-2-(7-methyl-1H-indol-4-yl)pyrimidin-4-yl)-3-methylmorpholine-3-carboxylic acid (125 mg, 0.337 mmol) in DMF (1.35 mL) was added trans-4-aminocyclohexanol hydrochloride (102 mg, 0.675 mmol) and the mixture stirred at 20 °C for 18 h. The reaction was poured into water (15 mL) and ethyl acetate (25 mL). The organic layer was separated and washed with 1 M aqueous sodium hydroxide, dried over magnesium sulfate, and concentrated in vacuo to give (S)-4-(5-fluoro-2-(7-methyl-1H-indol-4-yl)pyrimidin-4-yl)-N-((1r,4S)-4-hydroxycyclohexyl)-3-methylmorpholine-3-carboxamide (158 mg theoretical, yield determined on final compound) which was used without further purification. LRMS m/z: $[M+H]^+$ calcd for $C_{25}H_{30}FN_5O_3$, 468.2; found, 468.3.

15a. To a solution of (S)-4-(5-fluoro-2-(7-methyl-1H-indol-4-yl)pyrimidin-4-yl)-N-((1r,4S)-4-hydroxycyclohexyl)-3-methylmorpholine-3-carboxamide (158 mg, 0.337 mmol) in DMF (4 mL) was added 1 M NaHMDS solution in THF (1.34 mL, 1.34 mmol) and the solution stirred at 20 °C for 18 h. Glacial acetic acid (77 µl, 1.337 mmol) was added and the mixture was purified by prep HPLC (acidic mode). To remove TFA salt, the product was treated with 1 M aqueous lithium hydroxide (1 mL) and methanol (1 mL) for 15 min. The volatiles were removed in vacuo, and the residue stirred with water (1 mL) to form a precipitate which was collected by vacuum filtration, washed with cold water, and dried to give (S)-5-((1r,4S)-4-hydroxycyclohexyl)-6a-methyl-2-(7-methyl-1H-indol-4-yl)-6a,7,9,10-tetrahydro-[1,4]oxazino[3,4-h]pteridin-6(5H)-one (45 mg, 30% over 2 steps). LRMS m/z: $[M+H]^+$ calcd for $C_{25}H_{29}N_5O_3$, 448.2; found, 448.4. ^1H NMR (400 MHz, DMSO-d6) δ ppm 1.20 - 1.52 (m, 5 H) 1.67 (d, J=11.6 Hz, 2 H) 1.90 (br. s., 2 H) 2.22 - 2.38 (m, 1 H) 2.38 - 2.47 (m, 1 H) 2.53 (s, 3 H) 3.22 - 3.29 (m, 1 H) 3.45 - 3.56 (m, 1 H) 3.57 - 3.69 (m, 2 H) 3.97 (s, 1 H) 4.04 - 4.14 (m, 2 H) 4.27 (t, J=12.0 Hz, 1 H) 4.63 (br. s., 1 H) 6.99 (s, 1 H) 7.42 (s, 2 H) 8.02 (d, J=7.6 Hz, 1 H) 8.56 (s, 1 H) 11.20 (br. s., 1 H).

Cmpd 15b

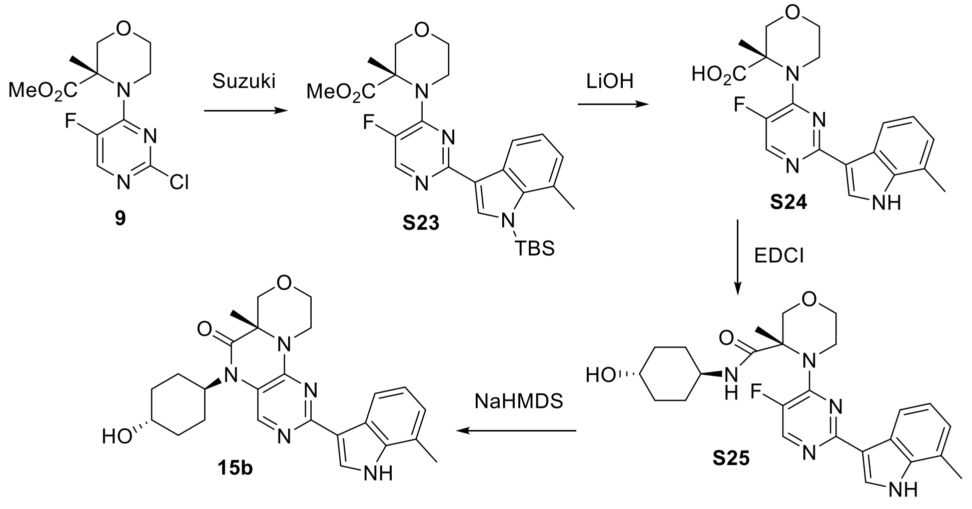

S23. A mixture of 1-(tert-butyldimethylsilyl)-7-methyl-3-(4,4,5,5-tetramethyl-1,3,2-dioxaborolan-2-yl)-1H-indole (4.75 g, 12.8 mmol), $PdCl_2(dppf)$ (234 mg, 0.32 mmol) and (S)-methyl 4-(2-chloro-5-fluoropyrimidin-4-yl)-3-methylmorpholine-3-carboxylate (1.85 g, 6.4 mmol) in 1,4-dioxane (21 mL) and

saturated aqueous sodium bicarbonate (11 mL) was heated in the microwave at 115 °C for 30 min. The solvent was removed in vacuo, and the residue was partitioned between water (50 mL) and ethyl acetate (50 mL). The mixture was filtered through Celite, and the layers separated. The aqueous layer was saturated with sodium chloride and extracted with ethyl acetate (2 x 50 mL). The combined organics were washed with brine (25 mL), dried with magnesium sulfate, and concentrated in vacuo. The crude residue was purified by silica gel chromatography eluted with 10-15% ethyl acetate/hexanes to give (S)-methyl 4-(2-(1-(tert-butyldimethylsilyl)-7-methyl-1H-indol-3-yl)-5-fluoropyrimidin-4-yl)-3-methylmorpholine-3-carboxylate (2.81 g, 88%) as a white solid. LRMS m/z: $[M+H]^+$ calcd for $C_{26}H_{35}FN_4O_3Si$, 499.3; found, 499.3.

S24. A solution of (S)-methyl 4-(2-(1-(tert-butyldimethylsilyl)-7-methyl-1H-indol-3-yl)-5-fluoropyrimidin-4-yl)-3-methylmorpholine-3-carboxylate (2.64 g, 5.39 mmol) in 1 M aqueous lithium hydroxide (52.4 mL, 52.4 mmol) and 1,4-dioxane (40 mL) was heated at reflux for 10 h. The volatile portion was removed in vacuo. The remaining aqueous layer was extracted with diethyl ether. The aqueous layer was acidified to pH ca. 5 which formed a white-yellow precipitate. The aqueous layer was extracted with ethyl acetate (3 x 50 mL). The organic layer was washed with brine (25 mL), dried with magnesium sulfate, and concentrated in vacuo to give (S)-4-(5-fluoro-2-(7-methyl-1H-indol-3-yl)pyrimidin-4-yl)-3-methylmorpholine-3-carboxylic acid (1.99 g, 99%) as a pale yellow solid. LRMS m/z: $[M+H]^+$ calcd for $C_{19}H_{19}FN_4O_3$, 371.1; found, 371.3. ^1H NMR (400 MHz, DMSO-d6) δ ppm 1.63 (s, 3 H) 2.47 - 2.49 (m, 3 H) 3.44 - 3.54 (m, 1 H) 3.70 (d, J=11.12 Hz, 1 H) 3.75 - 3.84 (m, 2 H) 3.89 - 4.00 (m, 2 H) 6.92 - 6.98 (m, 1 H) 6.98 - 7.06 (m, 1 H) 8.00 (d, J=3.03 Hz, 1 H) 8.26 (d, J=7.83 Hz, 1 H) 8.41 (d, J=6.57 Hz, 1 H) 11.59 (d, J=2.53 Hz, 1 H) 12.60 (s, 1 H).

S25. To a solution of HOBT (93 mg, 0.607 mmol), EDCI (116 mg, 0.607 mmol), DIPEA (141 µl, 0.810 mmol) and (S)-4-(5-fluoro-2-(7-methyl-1H-indol-3-yl)pyrimidin-4-yl)-3-methylmorpholine-3-carboxylic acid (150 mg, 0.405 mmol) in DMF (1.6 mL) was added trans-4-aminocyclohexanol hydrochloride (123 mg, 0.810 mmol) and the solution stirred at 20 °C for 18 h. The reaction was poured into water (15 mL) and ethyl acetate (25 mL). The layers were separated, and the organic layer was washed with 1 M aqueous sodium hydroxide, dried with magnesium sulfate, and concentrated in vacuo to give (S)-4-(5-fluoro-2-(7-methyl-1H-indol-3-yl)pyrimidin-4-yl)-N-((1r,4S)-4-hydroxycyclohexyl)-3-methylmorpholine-3-carboxamide (189 mg theoretical, yield calculated on final compound). LRMS m/z: $[M+H]^+$ calcd for $C_{25}H_{30}FN_5O_3$, 468.2; found, 468.3.

15b. To a solution of (S)-4-(5-fluoro-2-(7-methyl-1H-indol-3-yl)pyrimidin-4-yl)-N-((1r,4S)-4-hydroxycyclohexyl)-3-methylmorpholine-3-carboxamide (189 mg, 0.405 mmol) in DMF (4 mL) was added 1 M solution of NaHMDS in THF (1.34 mL, 1.34 mmol) and the solution was stirred at 20 °C for 18 h. More 1 M NaHMDS in THF (1.34 mL, 1.34 mmol) was added and the reaction stirred at 20 °C for 18 h. Glacial acetic acid (77 µl, 1.34 mmol) was added and the mixture was purified by prep HPLC (acidic mode). To remove the TFA salt, the product was treated with 1 M aqueous lithium hydroxide (1 mL) and methanol (1 mL) for 15 min. The volatiles were removed in vacuo and the residue stirred with water (1 mL). The precipitate was collected by vacuum filtration, washed with cold water, and dried to give (S)-5-((1r,4S)-4-hydroxycyclohexyl)-6a-methyl-2-(7-methyl-1H-indol-3-yl)-6a,7,9,10-tetrahydro-[1,4]oxazino[3,4-h]pteridin-6(5H)-one (19 mg, 10% over 2 steps) as a tan solid. LRMS m/z: $[M+H]^+$ calcd for $C_{25}H_{29}N_5O_3$, 448.2; found, 448.4. ^1H NMR (400 MHz, DMSO-d6) δ ppm 1.16 - 1.51 (m, 5 H) 1.65 (br. s., 2 H) 1.89 (br. s., 2 H) 2.20 - 2.44 (m, 2 H) 3.20 - 3.37 (m, 4 H) 3.43 - 3.70 (m, 3 H) 3.96 (br. s.,

1 H) 4.10 (br. s., 2 H) 4.25 (br. s., 1 H) 4.62 (br. s., 1 H) 6.90 - 7.08 (m, 2 H) 8.06 (br. s., 1 H) 8.31 (d, J=7.8 Hz, 1 H) 8.42 (br. s., 1 H) 11.55 (br. s., 1 H).

Cmpd 15c

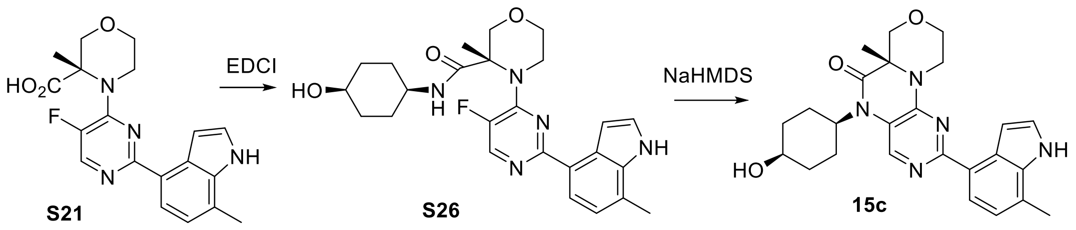

S26. To a solution of HOBT (78 mg, 0.506 mmol), EDCI (97 mg, 0.506 mmol), DIPEA (118 µl, 0.675 mmol) and (S)-4-(5-fluoro-2-(7-methyl-1H-indol-4-yl)pyrimidin-4-yl)-3-methylmorpholine-3-carboxylic acid (125 mg, 0.337 mmol) in DMF (1.35 mL) was added cis-4-aminocyclohexanol hydrochloride (102 mg, 0.675 mmol) and the mixture was stirred at 20 °C for 18 h. The reaction was poured into water (15 mL) and ethyl acetate (25 mL). The layers were separated, and the organic layer was washed with 1 M aqueous sodium hydroxide, dried with magnesium sulfate and concentrated in vacuo to give (S)-4-(5-fluoro-2-(7-methyl-1H-indol-4-yl)pyrimidin-4-yl)-N-((1s,4R)-4-hydroxycyclohexyl)-3-methylmorpholine-3-carboxamide (158 mg theoretical, yield calculated on final compound) which was used without further purification. LRMS m/z: $[M+H]^+$ calcd for $C_{25}H_{30}FN_5O_3$, 468.2; found, 468.3.

15c. To a solution of (S)-4-(5-fluoro-2-(7-methyl-1H-indol-4-yl)pyrimidin-4-yl)-N-((1s,4R)-4-hydroxycyclohexyl)-3-methylmorpholine-3-carboxamide (189 mg, 0.405mmol) in DMF (4 mL) was added 1 M NaHMDS in THF (1.34 mL, 1.34 mmol) and the solution stirred at 20 °C for 18 h. Glacial acetic acid (77 µl, 1.34 mmol) was added, and the mixture was purified by prep HPLC (acidic mode). To remove the TFA salt, the product was treated with 1 M aqueous lithium hydroxide (1 mL) and methanol (1 mL) for 15 min. The volatiles were removed in vacuo and the residue stirred with water (1 mL). The precipitate was collected by vacuum filtration, washed with cold water, and dried to give (S)-5-((1s,4R)-4-hydroxycyclohexyl)-6a-methyl-2-(7-methyl-1H-indol-4-yl)-6a,7,9,10-tetrahydro-[1,4]oxazino[3,4-h]pteridin-6(5H)-one (44 mg, 24% yield over 2 steps). LRMS m/z: $[M+H]^+$ calcd for $C_{25}H_{29}N_5O_3$, 448.2; found, 448.3. ^1H NMR (400 MHz, DMSO-d6) δ ppm 1.18 - 1.39 (m, 4 H) 1.40 - 1.70 (m, 3 H) 1.79 (d, J=12.4 Hz, 2 H) 2.52 - 2.71 (m, 5 H) 3.23 - 3.30 (m, 1 H) 3.57 - 3.74 (m, 2 H) 3.83 - 4.17 (m, 4 H) 4.44 - 4.66 (m, 2 H) 6.98 (d, J=7.8 Hz, 1 H) 7.42 (br. s., 2 H) 8.03 (d, J=7.1 Hz, 1 H) 8.56 (s, 1 H) 11.20 (br. s., 1 H).

Cmpd 15d

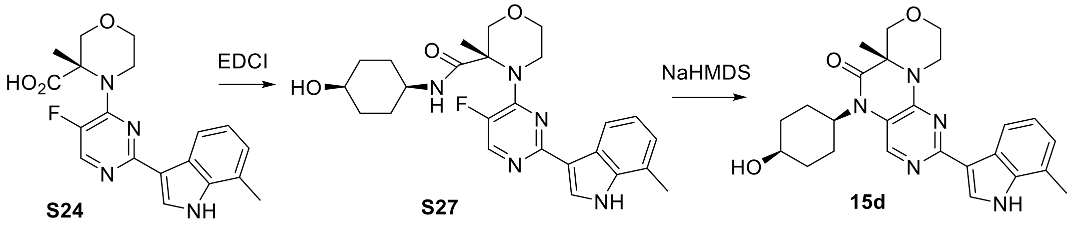

S27. To a solution of HOBT (93 mg, 0.607 mmol), EDCI (116 mg, 0.607 mmol), DIPEA (141 µl, 0.810 mmol) and (S)-4-(5-fluoro-2-(7-methyl-1H-indol-3-yl)pyrimidin-4-yl)-3-methylmorpholine-3-carboxylic acid (150 mg, 0.405 mmol) in DMF (1.6 mL) was added cis-4-aminocyclohexanol hydrochloride (123 mg, 0.810 mmol) and the mixture was stirred at 20 °C for 18 h. The reaction was poured into water (15 mL) and ethyl acetate (25 mL). The layers were separated and the organic layer was washed with 1 M aqueous sodium hydroxide, dried with magnesium sulfate, and concentrated in vacuo to give (S)-4-(5-fluoro-2-(7-methyl-1H-indol-4-yl)pyrimidin-4-yl)-N-((1s,4R)-4-hydroxycyclohexyl)-3-methylmorpholine-3-carboxamide (189 mg theoretical, yield calculated on final compound) which was used without further purification. LRMS m/z: $[M+H]^+$ calcd for $C_{25}H_{30}FN_5O_3$, 468.2; found, 468.3.

15d. To a solution of (S)-4-(5-fluoro-2-(7-methyl-1H-indol-3-yl)pyrimidin-4-yl)-N-((1s,4R)-4-hydroxycyclohexyl)-3-methylmorpholine-3-carboxamide (189 mg, 0.405mmol) in DMF (4 mL) was added 1 M NaHMDS in THF (1.34 mL, 1.34 mmol) and the solution stirred at 20 °C for 18 h. Glacial acetic acid (77 µl, 1.34 mmol) was added and the mixture was purified by prep HPLC (acidic mode). To remove the TFA salt, the product was treated with 1 M aqueous lithium hydroxide (1 mL) and methanol (1 mL) for 15 min. The volatiles were removed in vacuo and the residue stirred with water (1 mL). The precipitate was collected by vacuum filtration, washed with cold water, and dried to give (S)-5-((1s,4R)-4-hydroxycyclohexyl)-6a-methyl-2-(7-methyl-1H-indol-4-yl)-6a,7,9,10-tetrahydro-[1,4]oxazino[3,4-h]pteridin-6(5H)-one (32 mg, 18% yield over 2 steps) as a tan solid. LRMS m/z: $[M+H]^+$ calcd for $C_{25}H_{29}N_5O_3$, 448.2; found, 448.4. ^{1}H NMR (400 MHz, DMSO-d6) δ ppm 1.26 (br. s., 4 H) 1.39 - 1.68 (m, 3 H) 1.77 (br. s., 2 H) 2.57 - 2.68 (m, 1 H) 3.26 (br. s., 5 H) 3.64 (br. s., 2 H) 3.82 - 4.18 (m, 4 H) 4.57 (br. s., 2 H) 6.88 - 7.11 (m, 2 H) 8.08 (br. s., 1 H) 8.31 (br. s., 1 H) 8.44 (br. s., 1 H) 11.56 (br. s., 1 H).

Cmpd 15e

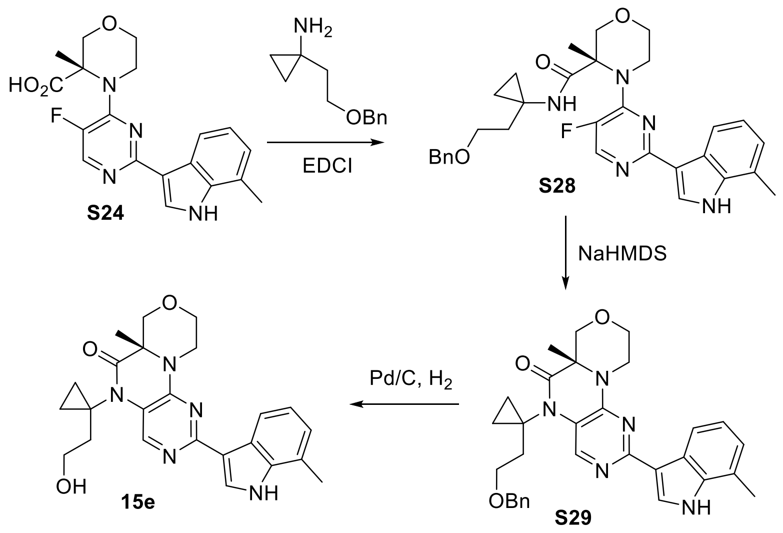

S28. A solution of (S)-4-(5-fluoro-2-(7-methyl-1H-indol-3-yl)pyrimidin-4-yl)-3-methylmorpholine-3-carboxylic acid (150 mg, 0.405 mmol), HOBT (62.0 mg, 0.405 mmol), EDC (155 mg, 0.810 mmol), 1-(2-(benzyloxy)ethyl)cyclopropanamine (194 mg, 1.012 mmol) and DIPEA (0.248 mL, 1.417 mmol) in DMF (2 mL) was stirred at 20 °C for 2 days. The solution was diluted with ethyl acetate (50 mL), washed with saturated aqueous ammonium chloride (50 mL) and brine (50 mL), dried with magnesium sulfate and concentrated in vacuo. The residue was purified by prep HPLC (basic mode) to give (S)-N-(1-(2-

(benzyloxy)ethyl)cyclopropyl)-4-(5-fluoro-2-(7-methyl-1H-indol-3-yl)pyrimidin-4-yl)-3-methylmorpholine-3-carboxamide (143 mg, 65%) as a yellow semisolid. LRMS m/z: $[M+H]^+$ calcd for $C_{31}H_{34}FN_5O_3$, 544.3; found, 544.4.

S29. To a solution of (S)-N-(1-(2-(benzyloxy)ethyl)cyclopropyl)-4-(5-fluoro-2-(7-methyl-1H-indol-3-yl)pyrimidin-4-yl)-3-methylmorpholine-3-carboxamide (143 mg, 0.263 mmol) in DMF (2630 µl) was added 1 M NaHMDS in THF (1.05 mL, 1.05 mmol) and the solution was stirred at 20 °C for 2 h. More 1 M NaHMDS in THF (0.26 mL, 0.26 mmol) was added and the solution stirred at 20 °C for 18 h. The solvent was removed in vacuo, and the residue stirred with water (5 mL). The precipitate was collected by vacuum filtration, rinsed with water, and dried to give (S)-5-(1-(2-(benzyloxy)ethyl)cyclopropyl)-6a-methyl-2-(7-methyl-1H-indol-3-yl)-6a,7,9,10-tetrahydro-[1,4]oxazino[3,4-h]pteridin-6(5H)-one (theoretical 138 mg, yield determined on final compound) which was used without further purification. LRMS m/z: $[M+H]^+$ calcd for $C_{31}H_{33}N_5O_3$, 524.3; found, 524.4.

15e. A slurry of (S)-5-(1-(2-(benzyloxy)ethyl)cyclopropyl)-6a-methyl-2-(7-methyl-1H-indol-3-yl)-6a,7,9,10-tetrahydro-[1,4]oxazino[3,4-h]pteridin-6(5H)-one (138mg, 0.264 mmol) and palladium on carbon (10 wt% Degussa) (28.0 mg, 0.264 mmol) in methanol (3 mL) and 4 M hydrochloric acid in 1,4-dioxane (0.527 mL, 2.108 mmol) at 20 °C was stirred under an atmosphere of hydrogen for 1 hour. The slurry was filtered with Celite to remove Pd/C and rinsed with methanol. The filtrate was concentrated in vacuo and triturated with cold methanol. The resultant precipitate was isolated by filtration to give (S)-5-(1-(2-hydroxyethyl)cyclopropyl)-6a-methyl-2-(7-methyl-1H-indol-3-yl)-6a,7,9,10-tetrahydro-[1,4]oxazino[3,4-h]pteridin-6(5H)-one (22 mg, 19% over 2 steps) as a white solid. LRMS m/z: $[M+H]^+$ calcd for $C_{24}H_{27}N_5O_3$, 434.2; found, 434.4. ^1H NMR (400 MHz, DMSO-d6, mixture of atropisomers) δ ppm 0.62 - 1.19 (m, 4 H) 1.27 - 1.37 (m, 3 H) 1.47 - 2.16 (m, 2 H) 2.51 (br s, 3 H) 3.21 - 3.32 (m, 1 H) 3.43 - 3.74 (m, 4 H) 3.88 - 4.02 (m, 1 H) 4.03 - 4.33 (m, 2 H) 4.34 - 4.59 (m, 1 H) 6.91 - 6.99 (m, 1 H) 7.00 - 7.10 (m, 1 H) 8.08 (d, J=2.8 Hz, 1 H) 8.32 (br d, J=7.8 Hz, 1 H) 8.46 - 8.56 (m, 1 H) 11.54 (br s, 1 H).

HPLC Chromatograms of in vivo compounds

Compound 1

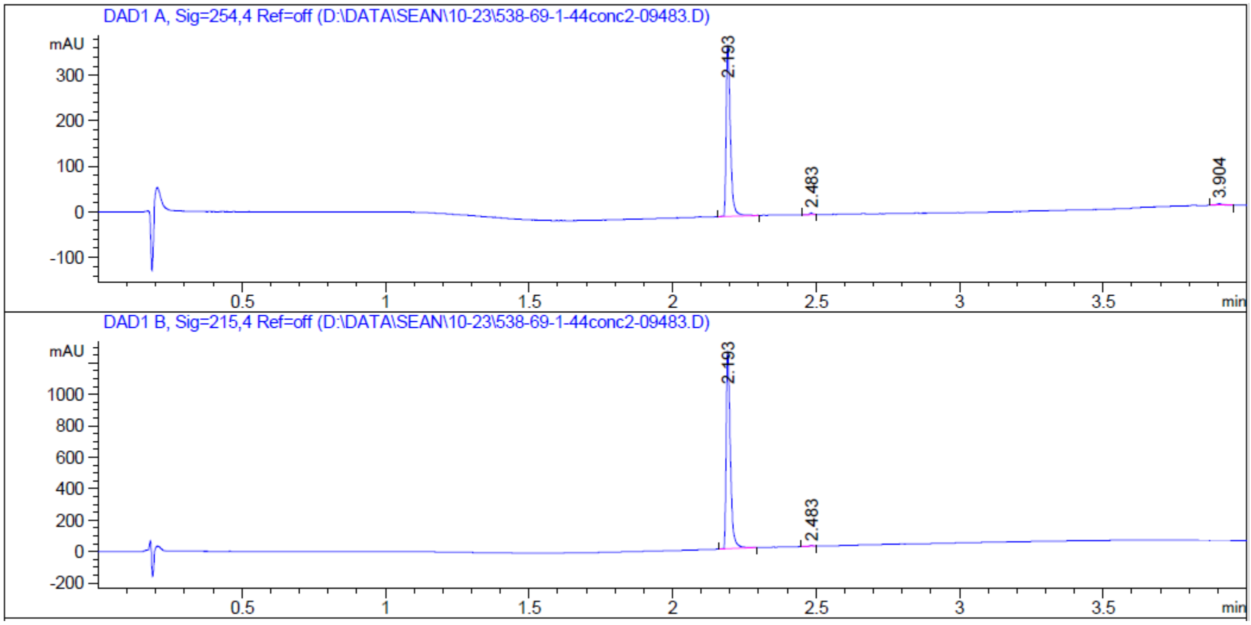

Signal 1: DAD1 A, Sig=254,4 Ref=off

Peak #	RetTime [min]	Type	Width [min]	Area [mAU*s]	Height [mAU]	Area %
1	2.193	BB	0.0158	385.38782	374.27420	97.9113
2	2.483	BB	0.0127	2.80612	3.28635	0.7129
3	3.904	BB	0.0216	5.41520	3.68844	1.3758
Totals :				393.60914	381.24899	

Signal 2: DAD1 B, Sig=215,4 Ref=off

Peak #	RetTime [min]	Type	Width [min]	Area [mAU*s]	Height [mAU]	Area %
1	2.193	BB	0.0156	1275.39001	1252.05579	99.5406
2	2.483	BB	0.0125	5.88659	6.87628	0.4594
Totals :				1281.27660	1258.93207	

Compound 4e

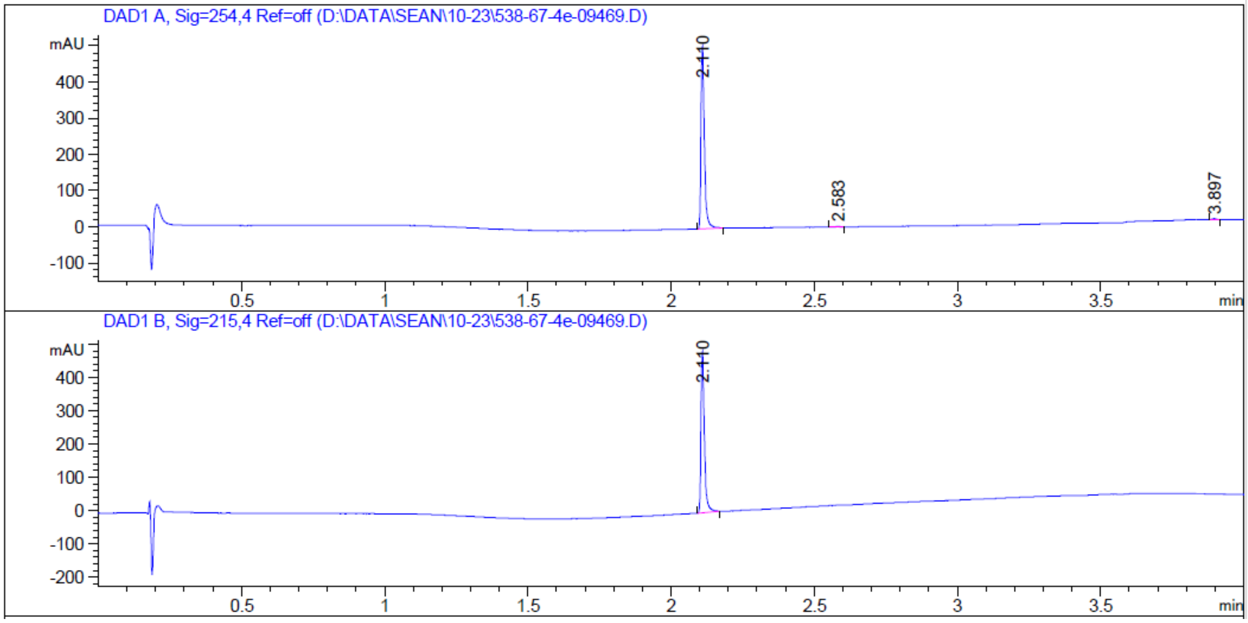

Signal 1: DAD1 A, Sig=254,4 Ref=off

Peak #	RetTime [min]	Type	Width [min]	Area [mAU*s]	Height [mAU]	Area %
1	2.110	BB	0.0130	423.10962	505.76749	98.9966
2	2.583	BB	0.0140	1.82828	1.93239	0.4278
3	3.897	BB	0.0137	2.46003	2.55679	0.5756
Totals :				427.39792	510.25667	

Signal 2: DAD1 B, Sig=215,4 Ref=off

Peak #	RetTime [min]	Type	Width [min]	Area [mAU*s]	Height [mAU]	Area %
1	2.110	BB	0.0130	403.96207	484.65436	100.0000
Totals :				403.96207	484.65436	

Compound 14b

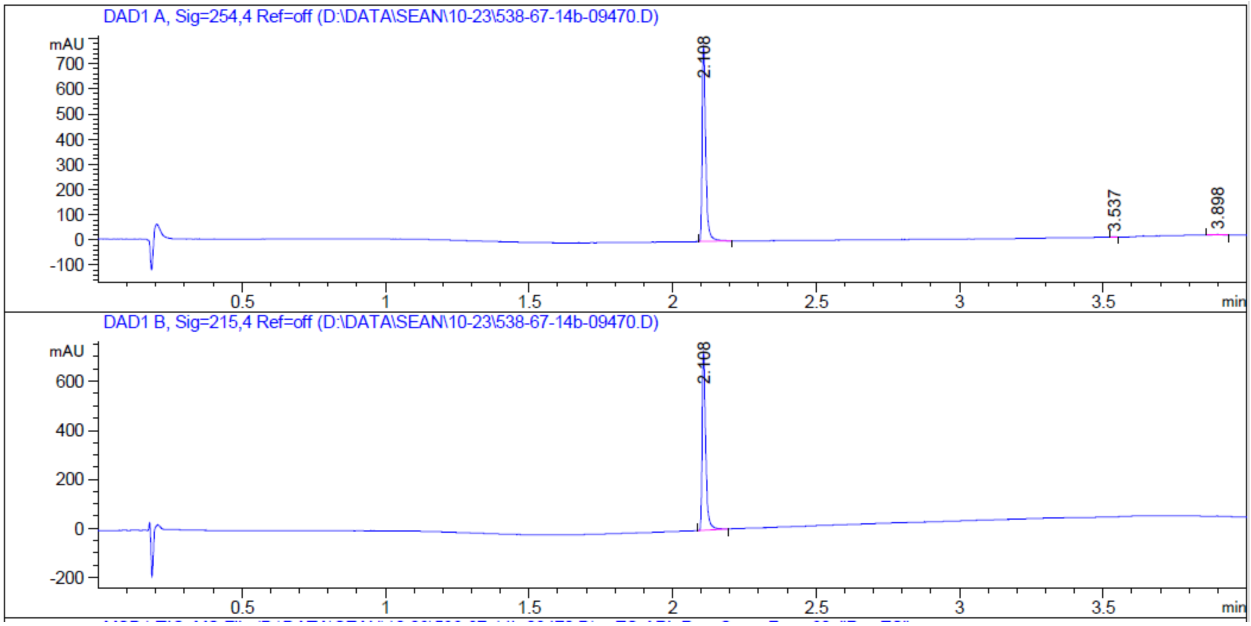

Signal 1: DAD1 A, Sig=254,4 Ref=off

Peak #	RetTime [min]	Type	Width [min]	Area [mAU*s]	Height [mAU]	Area %
1	2.108	BB	0.0135	681.12671	773.93384	98.9275
2	3.537	BB	0.0177	1.89148	1.64089	0.2747
3	3.898	BB	0.0246	5.49264	3.10772	0.7978
Totals :				688.51083	778.68244	

Signal 2: DAD1 B, Sig=215,4 Ref=off

Peak #	RetTime [min]	Type	Width [min]	Area [mAU*s]	Height [mAU]	Area %
1	2.108	BB	0.0135	638.59644	726.72394	100.0000
Totals :				638.59644	726.72394	

Compound 14c

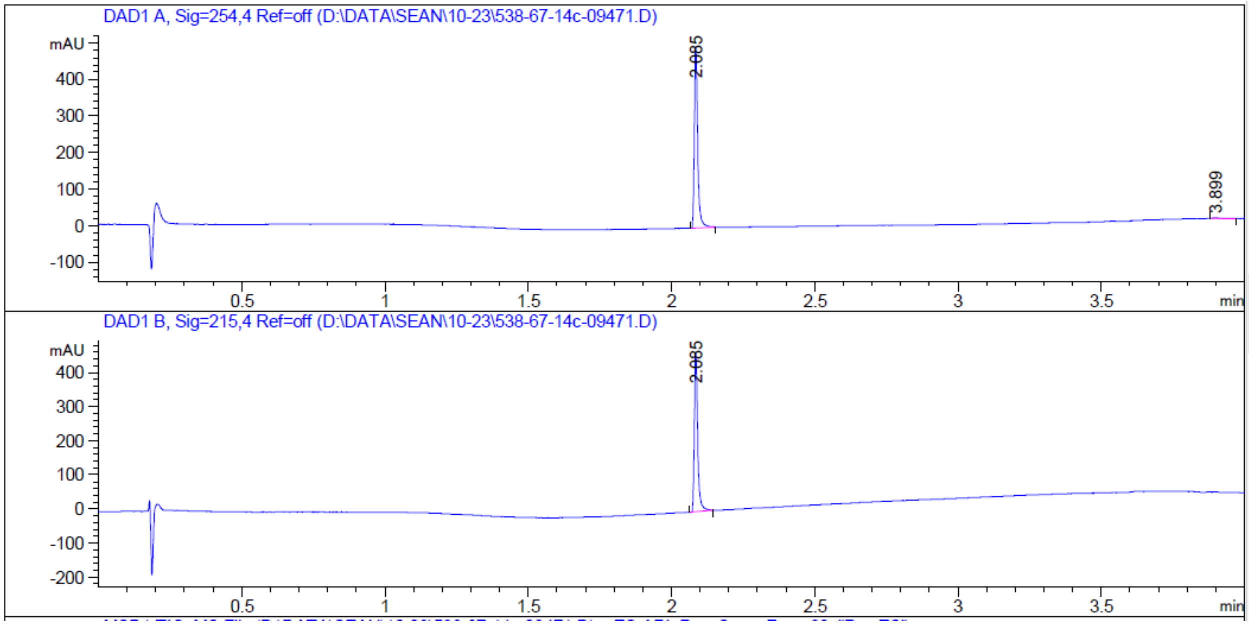

Signal 1: DAD1 A, Sig=254,4 Ref=off

Peak #	RetTime [min]	Type	Width [min]	Area [mAU*s]	Height [mAU]	Area %
1	2.085	BB	0.0124	402.84381	497.72684	99.1783
2	3.899	BB	0.0226	3.33753	2.06381	0.8217

Totals : 406.18134 499.79065

Signal 2: DAD1 B, Sig=215,4 Ref=off

Peak #	RetTime [min]	Type	Width [min]	Area [mAU*s]	Height [mAU]	Area %
1	2.085	BB	0.0122	378.86581	469.60449	100.0000

Totals : 378.86581 469.60449

Compound 15a

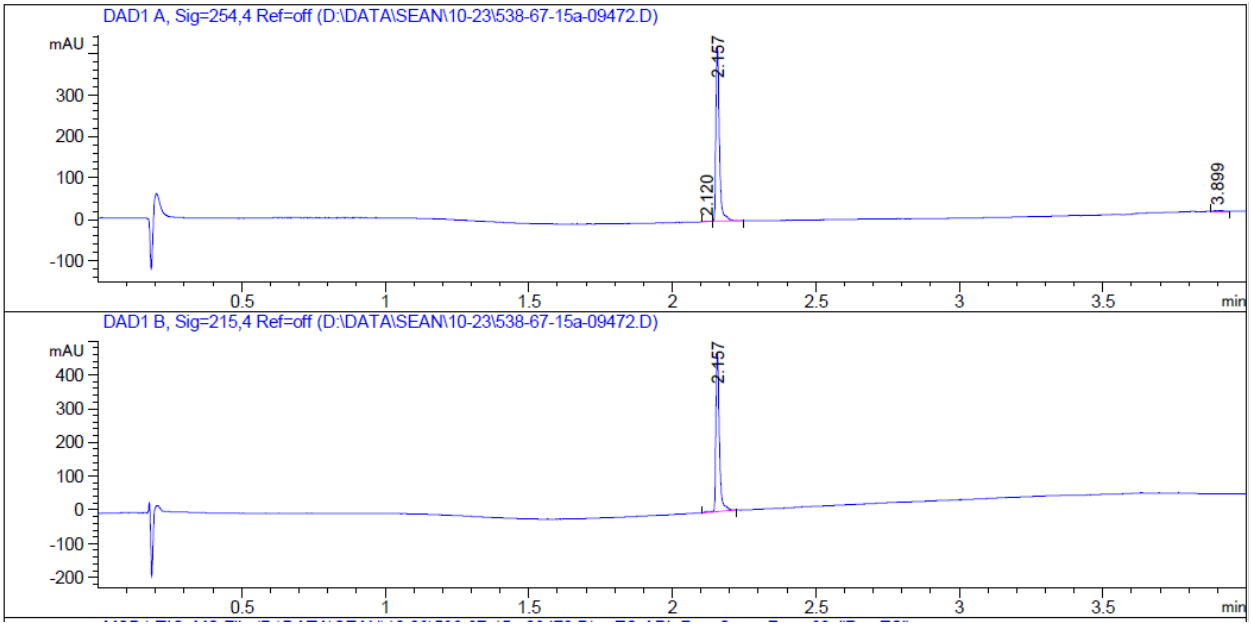

Signal 1: DAD1 A, Sig=254,4 Ref=off

Peak #	RetTime [min]	Type	Width [min]	Area [mAU*s]	Height [mAU]	Area %
1	2.120	BB	0.0159	2.20667	1.84920	0.6235
2	2.157	BB	0.0126	348.17950	422.13510	98.3786
3	3.899	BB	0.0202	3.53184	2.36161	0.9979
Totals :				353.91801	426.34591	

Signal 2: DAD1 B, Sig=215,4 Ref=off

Peak #	RetTime [min]	Type	Width [min]	Area [mAU*s]	Height [mAU]	Area %
1	2.157	BB	0.0127	391.49490	472.53519	100.0000
Totals :				391.49490	472.53519	

Cellular Assay Counts and Standard Deviations

Cmpd	Cell pEC50	N	pEC50 Std Dev
1	7.15	48	0.27
3a	7.42	6	0.15
3b	7.29	3	0.17
3c	7.04	3	0.14
4a	7.38	6	0.18
4b	7.21	1	
4c	6.98	2	0.23
4d	7.53	2	0.11
4e	7.67	13	0.24
4f	7.78	7	0.35
4g	7.59	1	
4h	7.32	1	
4i	8.06	3	0.20
13a	7.13	1	
13b	7.24	1	
13c	6.77	1	
13d	7.21	1	
13e	6.92	1	
14a	7.26	1	
14b	7.85	3	0.29
14c	7.9	11	0.26
15a	7.91	3	0.09
15b	8.15	1	
15c	7.75	1	
15d	8.04	1	
15e	8.34	1	

In Vitro Kinase Assays

Kinase Inhibition Assay

Materials:

FRAP1 (mTOR) recombinant protein and GFP-4E-BP1 were purchased from Invitrogen (Carlsbad, CA, US). The kinase activity assay was conducted in 50 mM Hepes buffer containing 10 mM $MgCl_2$, 0.01% Brij, 0.2 mM EDTA, 2 mM DTT and 1% DMSO (after compound addition) at pH 7.3.

Methods:

The IC_{50} value for each compound was determined in the presence of compound (various concentrations, from 0 to 10 µM) and a fixed amount of ATP (50 µM, final concentration), substrate GFP-4E-BP1 (400 nM, final concentration). The enzymatic reaction was initiated by adding FRAP1 (mTOR) (2 nM, final concentration). The assay was conducted at room temperature (~22 °C). After 30 min, the enzymatic reaction was stopped using Tb-anti-p4E-BP1 (2 nM final concentration, Invitrogen) and EDTA (20 mM final concentration, Sigma) in TR-FRET dilution buffer provided by Invitrogen. All the reagents were dispensed using a Multidrop Combi reagent dispenser (ThermoFisher Scientific, Waltham, MA US) into black 384 SV Greiner plates. The release of product was detected using a BMG PHERAstar plate reader (BMG LABTECH, Ortenberg, Germany) and the Lantha Screen module (337 nm excitation wavelength) and measuring the ratio of fluorescence 520/490 nm (emission wavelengths). Experimental data was fitted using Eq. (1):

$$V_i/V_o = 100 / (1 + (I/IC_{50})^n) \quad (1)$$

V_i and V_o are the rates of the enzyme activity in the presence and in the absence of inhibitor; n is the Hill coefficient; I is the free inhibitor concentration; IC_{50} is a measure of potency that is equivalent to a concentration of inhibitor that leads to a 50% inhibition of the enzyme activity.

Cellular p-AKT (ser473) assay in PC-3 cells

PC-3 cells (PTEN deleted, human prostate cell line) were maintained in F12K media with 10% heat-inactivated fetal bovine serum (FBS) in 37 °C, 5% CO_2 incubator. This method describes the procedure for a p-AKT (ser473) AlphaScreen assay in PC-3 cells. p-AKT (ser 473) assay is an AlphaLISA SureFire kit from Perkin Elmer (catalog# TGRAS500). On day 1, 50,000 PC-3 cells were plated in 160 µL complete media per well in 96-well tissue culture plate (Corning catalog#3595). Plates were placed in 37 °C, 5% CO_2 incubator for 24 h. On day 2, all media was removed and replaced with 160 µL starvation media which is F12K media (no FBS) and 0.1% BSA. Plates are placed in 37 °C, 5% CO_2 incubator for 16 h. On day 3, compounds were both solubilized and serially diluted in DMSO and added to a secondary plate containing starvation media. 40 µL of compound/starvation media were added to the 160 µL in cell plate and plates were placed back in the incubator for 1 h. Cells were then activated by adding 22 µL FBS (for a final concentration of 10% FBS) for 10 min in the incubator. Media was removed from the well and cells were lysed in 100 µL SureFire kit lysis buffer and then shaken for 15 min at 900 rpm. AlphaScreen

assay was performed according to manufacturer's instructions. 4 µL of lysate was added to PerkinElmer 96-well Proxiplate then 5 µL of Reaction Buffer plus Activation Buffer. Plates were shaken for 2 h at room temperature. 2 µL of Donor Beads in Dilution Buffer were added to the plate and the plate was shaken for 2 h at room temperature. The plate was read using BMG Labtech PHERAstar using AlphaScreen settings. Each analog was run in duplicate wells and a single IC_{50} value was calculated in IDBS ActivityBase.

In vivo Xenograft Model

All animal studies were conducted in accordance with Takeda California Institutional Animal Care and Use Committee guidelines in a facility accredited by the American Association for Accreditation of Laboratory Animal Care. Female Nu/Nu mice (Balb/c), approximately 28 days of age and weighing 20 to 23 grams were obtained from Harlan Laboratories. Immuno-compromised and/or inbred mice are used extensively for in vivo cancer research for detecting pharmacological capabilities induced by anti-cancer agents and therefore were chosen as the most appropriate animal model for this study. Five million A549 cells/animal (0.2 mL) w/ Matrigel were implanted S.C. into the right flank of Balb/c Nu/Nu mice. Low passage A549 cells were grown in F12 media and supplied at a concentration of 50 million cells/mL in DPBS. Treatment was initiated when all study animals had small tumors averaging approximately 150-180 mg. Dosing solutions/suspensions were prepared every 5 days. Compound **1** was suspended in 30% Captisol in 0.05 M MSA pH ~1.5. Compound **14c** was suspended in 0.5% MC. Dose solutions/suspensions were given orally at a dose volume of 5 mL/kg. Animals in the control groups received 0.5% MC vehicle in a dose volume equivalent to drug-treated animals. Therapy consisted of oral gavage administration of compound **1** at 30 milligrams drug per kilogram animal weight (mpk) or compound **14c** at either 2 or 6 mpk. Each dosing group had five mice and were dosed QD on days 1 through 14. The general health of the mice was monitored, and mortality was assessed daily. Tumor dimensions were recorded 2 times per week starting one day before the first day of treatment. Toxicity was assessed as net body weight loss and lethality. Tumor weights are calculated using the equation $(a \times b^2)/2$, where a and b refer to the larger and smaller dimensions collected at each measurement. Error bars in each plot of % tumor volume change vs. days of treatment were calculated as the standard error of the means.

Tumor Growth Inhibition Body Weights

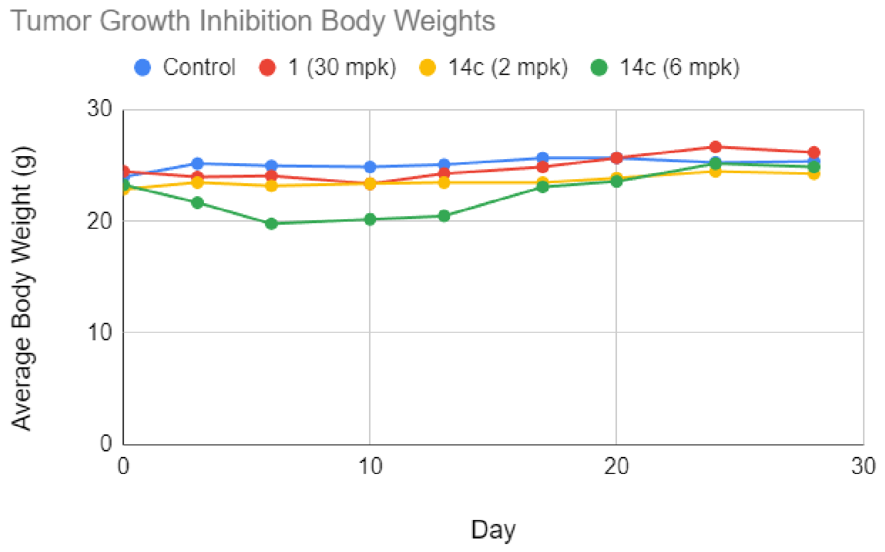

Supplement: Supplementary file 1 — ml3c00351_si_001.pdf [file ml3c00351_si_001.pdf]
